# Effect of creep-feeding supplementation during the pre-weaning phase on gene co-expression in *Longissimus thoracis* muscle of F1 Angus x Nellore calves at weaning

**DOI:** 10.1371/journal.pone.0339043

**Published:** 2025-12-18

**Authors:** Henrique Gonçalves Reolon, João Pedro Martins Borges, Gabriel Luiz Navarro Liberal, German Dario Ramírez-Zamudio, Guilherme Luis Pereira, Juliana Akamine Torrecilhas, Welder Angelo Baldassini, Marcos Eli Buzanskas, Otávio Rodrigues Machado Neto, Luis Artur Loyola Chardulo, Rogério Abdallah Curi

**Affiliations:** 1 São Paulo State University (UNESP), School of Agricultural and Veterinary Sciences (FCAV), Jaboticabal, São Paulo, Brazil; 2 São Paulo State University (UNESP), School of Veterinary Medicine and Animal Science (FMVZ), Botucatu, São Paulo, Brazil; 3 University of São Paulo (USP), School of Animal Science and Food Engineering, Pirassununga, São Paulo, Brazil; 4 Center for Nutrition and Pregnancy, Department of Animal Sciences, North Dakota State University, Fargo, North Dakota, United States of America; 5 University of Guelph, Ontario Agricultural College, Guelph, Ontario, Canada; University of Agricultural Sciences, INDIA

## Abstract

The aim of this study was to analyze gene co-expression in skeletal muscle of calves with or without creep-feeding during the pre-weaning phase. Forty-eight F1 uncastrated male Angus-Nellore calves were divided into two groups: G1 - no creep-feeding, and G2 - creep-feeding. After weaning (210 days), all animals were kept in the feedlot for 180 days under the same conditions. Weaning weight, backfat thickness, and intramuscular fat content were significantly higher in G2, with intramuscular fat and marbling score being 17.2% and 14.0% higher, respectively, compared with G1 (P < 0.05). *Longissimus thoracis* muscle samples were collected at weaning for transcriptome analysis (RNA-Seq) in 12 animals of each group. Gene co-expression analysis using the CEMiTool R package identified seven modules; five showed differential activity between groups (adjusted P < 0.002). Modules 1, 2, and 3 showed the greatest association with treatments. Hub genes and enrichment in biological pathways and processes were identified in these modules. The absence of supplementation was associated with increased connectivity of hub genes involved in insulin signaling, oxidative metabolism, and cell cycle regulation, including *CDKN1A*, *FOXO1*, and *NAMPT*. These genes were enriched in processes related to lipid oxidation and response to ketone bodies, suggesting reduced myogenic and adipogenic activity. Notably, FOXO1 has context-dependent effects on adipogenesis, acting as both an inducer and inhibitor depending on the differentiation stage. In contrast, supplementation increased the activity of hub genes involved in cell signaling, muscle development, and differentiation, such as *ITGB6*, *REEP1*, *TPCN1*, *PPARA*, *PTPN11*, and *MAP3K20*, and enriched processes associated with muscle and adipose cell development. In conclusion, creep-feeding supplementation during the pre-weaning phase altered gene co-expression in skeletal muscle, activating pathways related to myogenesis, adipogenesis, and energy metabolism. These results suggest potential lasting molecular effects consistent with increased intramuscular fat deposition during finishing.

## 1 Introduction

Among the sensory attributes of beef, tenderness is widely recognized as the most influential factor for consumer acceptance of the product. Classical studies have indicated this attribute to be the most valued, with consumers even being willing to pay higher prices for more tender meat [1–3]. More recent studies reinforce this perception, associating tenderness with higher quality cuts, generally identified as prime products, and with a greater purchase intention [4,5]. In addition to tenderness, intramuscular fat (marbling) has become a central attribute in premium beef markets due to its contribution to juiciness, flavor, and overall sensory quality [6–8].

Intramuscular fat deposition is influenced by different factors, with genetics and breed being the most determinant [9,10], followed by nutritional strategies [11]. In Brazil, industrial crossbreeding is being widely employed to improve productive performance by exploiting the effects of heterosis [12,13]. Among British breeds, Angus cattle are particularly notable for their contribution to enhancing carcass and meat quality traits when crossed with zebuine animals, thereby facilitating access to premium markets [13]. Furthermore, feedlot finishing is a consolidated strategy to increase productivity and improve the quality of carcasses and meat; this strategy accounts for approximately 17% of slaughters in Brazil [14–16].

The nutritional and breeding history of animals can significantly affect their performance during the finishing phase, as well as final carcass and meat quality [17,18]. Within this context, creep-feeding, a practice that consists of providing supplemental feed to calves in pasture systems from the first months of life until weaning, has been extensively studied in Brazil and other countries. This practice has shown positive impacts on the weight gain of animals [19–22]. In addition to improving performance during the early and finishing phases, creep-feeding can have a positive impact on meat quality traits such as intramuscular fat deposition. Previous results published by our research group using this nutritional practice in crossbred Angus × Nellore calves revealed differences in the expression of genes related to adipogenesis and energy metabolism in skeletal muscle, which contribute to a long-term increase in intramuscular fat deposition [23].

Within this context, the use of omics tools is a promising approach to investigate how early nutritional strategies modulate gene expression in target tissues such as skeletal muscle. There have been considerable advances in molecular and bioinformatics approaches in recent years, which offer great potential for elucidating biological processes and metabolic pathways regulated by genes of economic interest to the meat industry [24,25]. Recent approaches such as gene co-expression analyses play a fundamental role by enabling the identification of gene networks that act in a coordinated manner, thereby contributing to a deeper understanding of the mechanisms that regulate productive traits [26,27]. Such analyses do not only confirm genes previously identified as differentially expressed but also reveal other genes that, despite the lack of significant differences in expression between the groups analyzed, play important roles in the regulation of phenotypes of interest [28].

We hypothesized that creep-feeding supplementation during the pre-weaning phase would increase the activity of gene modules related to myogenesis and adipogenesis in the *Longissimus thoracis* muscle of F1 Angus × Nellore calves. Therefore, the aim of this study was to analyze the effects of creep-feeding supplementation during the pre-weaning phase on gene co-expression profiles in skeletal muscle of Angus × Nellore calves.

## 2 Materials and methods

### 2.1 Animals and treatments

This study was conducted in strict accordance with the recommendations of the Brazilian National Council for the Control of Animal Experimentation (CONCEA). The protocol was approved by the Animal Use Ethics Committee of the School of Agricultural and Veterinary Sciences (FCAV), São Paulo State University “Júlio de Mesquita Filho” (Unesp), Jaboticabal Campus, Brazil (Protocol Number: 013689/19). Muscle biopsies were performed under local lidocaine anesthesia, and animals were humanely slaughtered by penetrating captive bolt stunning followed by exsanguination.

This study used 48 uncastrated crossbred F1 Angus × Nellore calves with a mean body weight of 59.4 ± 2.5 kg, born to Nellore cows (*Bos indicus*) inseminated by the same Aberdeen Angus bull (*Bos taurus*). During the pre-weaning phase, between 30 days of age and weaning (approximately 210 days), the calves were divided into two experimental groups of 24 animals each: group 1 (G1), control group not submitted to creep-feeding supplementation; group 2 (G2), submitted to creep-feeding supplementation. During this period, G1 and G2 calves were kept together with their respective mothers in two separate *Urochloa* spp. paddocks of 20 hectares each.

Calves in G2 had exclusive access to a supplement fed (Table 1) at an amount equivalent to approximately 1% of their body weight, which was adjusted weekly based on body weight measurements. After weaning, calves in the two groups were confined for approximately 180 days. The animals were housed in covered collective pens with concrete floors, with a capacity of three animals per pen and an area of 10 m^2^ per calf. Both groups received the same diet (Table 2), which was formulated with the RLM 3.3 (Ração de Lucro Máximo) software[29] based on the ESALQ-Tropicalized NRC system. The diet was offered *ad libitum* in two daily meals (8:00 a.m. and 4:00 p.m.), and feed refusals were collected and weighed daily to calculate intake and adjust feed supply.

**Table 1:** Ingredients and chemical‐bromatological composition of supplement fed.

Ingredients	(g/Kg of DM)
Ground corn	448.0
Soybean meal	404.0
Mineral mix	148.0
**Chemical composition**	**(g/Kg of DM)**
Crude Protein	220.0
Total Digestible Nutrients	650.0

DM = Dry Matter.

**Table 2:** Ingredients and chemical‐bromatological composition of the feedlot diet.

Ingredients	(g/Kg of DM)
Sugarcane bagasse	90.0
Tifton‐85 hay	30.0
Ground corn	660.4
Soybean meal	175.3
Urea	3.8
Mineral‐vitaminic supplement	37.4
**Chemical composition**	**(g/Kg of DM)**
Crude Protein	153.4
Neutral Detergent Fiber	256.8
Ether Extract	35.3

DM = Dry Matter.

Weights were recorded during different phases of the experiment to assess animal performance. Body weight was measured after a 16-hour fast at the beginning of the pre-weaning phase (initial weight - BWi), at the end of weaning (weaning weight – WW), and at the end of the feedlot period (final weight - BWf). Based on these measurements, the average daily weight gain (ADG) was calculated as follows: ADG1, beginning of the pre-weaning phase to weaning (BWi to WW), and ADG2, postweaning period to the end of the feedlot period (WW to BWf). The results were published previously by [23].

### 2.2 Muscle tissue sampling

At weaning, a subsample of 12 calves of each treatment were randomly selected for collection of skeletal muscle samples by biopsy for RNA sequencing. The TOST procedure (Two One-Sided Tests) was implemented using the TOSTER package [30,31] of the R software (v.4.3.2; [32]), adopting equivalence bounds of ±1 standard deviation, where P eq < 0.05 indicated statistical equivalence between the sampling and subsampling within each group.

Skeletal muscle biopsies were collected from the *Longissimus thoracis* (LT) between the 12^th^ and 13^th^ thoracic vertebrae. Animals were restrained in a squeeze chute to minimize stress and movement. First, the region was shaved and asepsis was performed. Next, 4 mg/kg body weight of 2% lidocaine was administered subcutaneously without epinephrine (Lidovet, Laboratório Bravet, Rio de Janeiro, RJ, Brazil). Adequate local anesthesiafoxo was confirmed by lack of response to pin-prick before incision. A 1 cm incision was made and approximately 1 g of muscle tissue was collected with a sterile Bergström biopsy needle (Ortovet, Belo Horizonte, MG, Brazil). The material was immediately stored in liquid nitrogen and later transferred to an ultrafreezer for storage at −80°C.

### 2.3 Evaluation of carcass and meat quality traits

After the finishing period, all animals were fasted from solids for 16 hours (with free access to water) and transported to a commercial slaughterhouse located 120 km from the feedlot. Upon arrival, the animals were unloaded and held in lairage with free access to water for 12 hours before slaughter. Humane slaughter procedures were followed, with animals stunned using a penetrating captive bolt and immediately exsanguinated, in accordance with the guidelines for humane slaughter established by the Regulation for the Sanitary Inspection and Industrialization of Products of Animal Origin [33].

The carcasses were identified, washed, and divided into two halves. Each half-carcass was weighed individually to obtain the hot carcass weight (HCW) and then kept in a cold room for approximately 24 h at 1 °C. After cooling, the carcasses were removed and weighed again. From the left half-carcass, the *Longissimus thoracis* (LT) muscle was separated, and ribeye area (REA) and backfat thickness (BFT) were measured between the 12^th^ and 13^th^ ribs before deboning, following USDA-AMS anatomical and procedural guidelines [34], with adjustments using digital quantification of the traced muscle area in ImageJ® software (National Institutes of Health, Bethesda, Maryland, USA). Backfat thickness was measured with a precision caliper at three-fourths of the medial border of the LT muscle.

Beef samples (sirloin steaks) were then collected from the left half-carcass, between the 12th and 13th thoracic vertebrae, in the cranial direction. The samples were vacuum-packed and stored until laboratory analysis of physicochemical quality traits. The following attributes were evaluated: marbling score (MS), intramuscular fat content expressed as total lipid percentage (%F), and Warner–Bratzler shear force (WBSF) after 7 and 14 days of aging (WBSF7 and WBSF14). The MS was assessed by a single, trained evaluator using the Brazil Beef Quality standards (https://www.brazilbeefquality.com/), which provide a scale from 100 to 1100, adapted from AUS-MEAT, where higher values indicate greater marbling. The intramuscular fat content was determined by infrared spectroscopy using a FoodScanTM (Foss NIRSystems, Laurel, MD, USA). Warner-Bratzler shear force was determined using the procedures described by AMSA [35] and [36] with minor adaptations. In brief, samples were placed on a rack attached to a glass baking dish and baked in an industrial electric oven (Feri90 Venâncio, Venâncio Aires, RS, Brazil). When the internal temperature of the steaks reached 40 °C, they were turned over and left in the oven until the final temperature reached 71 °C. The internal temperature was monitored using thermocouples (ThermoPro Model TP-16, Hong Kong, China). After cooking, the samples were cooled, weighed, and refrigerated at 4 °C for 24 h. To determine WBSF, eight cores with a diameter of 1.27 cm were sectioned using a Brookfield CT-3 Texture Analyzer (Ametek Brookfield, Middleborough, MA, USA). The device was equipped with a 3.07-mm-thick stainless-steel blade with a V-shaped cutting edge (60° angle) and a 25 kg load cell, operated at a speed of 20 cm/min. The results were expressed in kilograms (kg) and were the average of eight repetitions (cylinders) per sample, excluding the highest and lowest values to minimize variability.

### 2.4 Statistical analysis

The zootechnical performance, carcass, and meat quality data (BWi, WW, ADG1, BWf, ADG2, HCW, REA, BFT, MS, %F, WBSF7, and WBSF14) were previously analyzed and published in [23]. Briefly, the data from 48 animals (n = 24/group) were evaluated for the presence of outliers, homogeneity of variances, and normality of residuals. All variables met the assumptions of parametric statistics, and no transformations were required. Results were expressed as means and standard errors, and comparisons between groups were performed using the Student’s t-test, adopting a significance threshold of *P* < 0.05. Analyses were conducted in R software (v.4.2.1; [32]).

The Cohen’s d standardized effect size was calculated using the effectsize package [37] of the R software (v.4.3.2; [32]), based on group means and standard deviations, and effects were interpreted following [38] conventions.

### 2.5 RNA extraction and sequencing

Total RNA was individually extracted from 100 mg LT muscle samples using TRIzol® (Life Technologies, USA, Cat. no. 15596018) according to the manufacturer’s instructions. RNA quality was analyzed in the Bioanalyzer 2100® (Agilent, USA). RNA concentration was determined with the Qubit RNA HS Assay Kit (Thermo Fisher Scientific, USA, Cat. no. Q32855).

From the total RNA obtained, an aliquot of 2 µg (0,002 mg) per sample was used as input for library construction with the TruSeq Stranded mRNA Library Prep Kit (Illumina, USA, Cat. no. 20020594). This protocol includes poly(A) selection with oligo-dT beads, an on-bead DNase treatment performed to remove potential genomic DNA contamination, and heat-mediated RNA fragmentation at (94°C for 8 min) prior to first- and second-strand cDNA synthesis, end-repair, A-tailing, indexed adapter ligation, and limited-cycle PCR amplification for 15 cycles. The mean size of the libraries was estimated in the Bioanalyzer 2100® (Agilent, USA), and quantitative PCR (RT-qPCR) was applied to quantify the libraries using the KAPA Library Quantification kit (KAPA Biosystems, USA, Cat. no. KK4824). Clustering and sequencing were performed on a single lane using the HiSeq2500 v4 2x100 bp kit (Illumina, USA, Cat. no. FC-401–4002) to produce 100-bp paired-end (PE) reads, with a minimum coverage of 16 million reads per sample.

### 2.6 Mapping of sequences to the reference genome

The sequencing data generated on the Illumina HiSeq System platform were converted into FastQ format and separated by library (multiplexed data) using Casava 1.8.2 (Illumina, USA). The FastQC v.0.11.9 software [39] was used to analyze the quality of raw reads. Adapter sequences and low-quality sequences were removed using Fastp v.0.23.1 [40], with the following parameters: automatic adapter detection, trimming of bases with Phred score < 20, minimum read length of 50 bp, removal of reads with more than 5% ambiguous bases (N), and filtering to retain at least 30% of high-quality bases per read. After this step, the quality of the reads was reassessed by combined visualization of all FastQC outputs using the MultiQC v.1.13 program [41] to confirm the increase in quality. Next, the reads were mapped to the bovine reference genome (*Bos taurus* – ARS-UCD1.3), available at http://www.ensembl.org/Bos_taurus/Info/Index/ (accessed August 19, 2021), using STAR v.2.7.20 [42] with default parameters, including a maximum mismatch ratio of 0.04, intron size between 20 and 1,000,000 bp, and up to 20 multiple alignments allowed per read.

Mapping was performed independently for each sample/library. A file with.bam extension was generated for each library, which contained the alignment of the fragments to the reference genome. The mapped reads were counted using featurecounts v.2.0.3 [43]. Only PE reads mapped to a unique location in the genome (uniquely PE reads) were used in the gene co-expression analyses.

### 2.7 Determination of co-expression modules

For the normalization of raw count data prior to co-expression analysis, lowly expressed genes were first filtered out by retaining only those with counts per million (CPM) ≥ 1 in at least the number of samples corresponding to the smallest experimental group. Normalization of library size factors was then performed using the trimmed mean of M-values (TMM) method implemented in the edgeR package [44]. Since the CEMiTool v. 1.28.0 package [45] requires variance-stabilized data for module detection, the normalized matrix was additionally subjected to a variance stabilizing transformation (VST), ensuring homoscedasticity of gene expression values across the full dynamic range.

This processed matrix, together with the required input files, was then provided to CEMiTool for the inference of co-expressed gene modules. The input files included: (i) count matrix (gene transcripts x samples); (ii) sample annotation file, which indicated the distribution of samples within each treatment; (iii) file of gene sets of canonical pathways from the Kyoto Encyclopedia of Genes and Genomes (KEGG), WikiPathways (WP), and Reactome databases and genes derived from the biological processes of the gene ontology (GOBP) database, all available at https://www.gsea-msigdb.org/gsea/msigdb/; (iv) file of gene/protein interactions (interactome) for *Bos taurus* obtained from the STRING 12.0 database (interaction data - https://string-db.org/cgi/download).

In the first step, the package automatically filtered the input genes. Assuming an inverse gamma distribution of the genes, selection was performed considering p = 0.01. In addition, the variance-stabilizing transformation (VST) filter was applied. By default, the package also removes 25% of the genes with the lowest average expression in the samples [45]. In the next step, Pearson’s correlation coefficients between each pair of genes present in the expression file were determined.

The network_type parameter of the package was changed from unsigned to signed. This parameter was used because, according to [46], an unsigned network considers absolute correlation values, i.e., two genes with a negative correlation are considered to be co-expressed. However, this negatively correlated gene is also positively co-expressed with other different genes, which are grouped in the same module. Thus, according to the authors, a signed network in which correlation values are scaled from 0 to 1 solves this problem and creates networks in which biologically significant modules are better separated. The set of parameters applied in the CEMiTool R package are shown in Table 3.

**Table 3:** Parameters applied in the CEMiTool R package for co-expression analysis.

Parameter	Description	Value
apply_vst	–	TRUE
beta	Value of beta chosen	16
cor_method	Correlation method	pearson
diss_thresh	Dissimilarity threshold to be used as cutoff on hierarchical clustering	0.8
filter	–	TRUE
filter_pval	p-value used on filtering	0.1
merge_similar	Should similar modules be merged?	TRUE
min_ngen	Minimum number of genes per module	30
network_type	–	signed
n_mods	Number of modules returned by CEMiTool	7
phi	Area under curve/total area in the beta vs R squared graph	0.656
r2	Determination coefficient. Reflects the “scale-freeness” of the resulting network	0.84

### 2.8 Complementary analyses

Gene Set Enrichment Analysis (GSEA) was performed for each co-expression module using the GSEA algorithm of the CEMiTool. This analysis permitted to identify the collective association of genes of one module with the treatments applied in the study [47].

Overrepresentation analysis was performed for KEGG, Reactome and WikiPathways metabolic pathways, in addition to GO biological processes, using the clusterProfiler v. 4.10.1 [48] and enrichplot v. 1.22.0 packages in R. Terms that had false discovery rate (FDR)-adjusted p-values ≤ 0.05 were defined as significantly enriched. This analysis permitted to determine whether the genes in each module were enriched in known pathways or processes.

Co-expression networks generally include modules with many genes; thus, prospecting of genes within each module that best represent its behavior is essential. For this purpose, hub genes were identified. Interactions in the modules, which are the result of the integration of co-expression networks with interactome data, were identified by CEMiTool; based on this analysis, the package returned the main hub genes (genes with the largest number of connections within each module).

## 3 Results

### 3.1 Performance and carcass and meat quality

**Fig 1:**
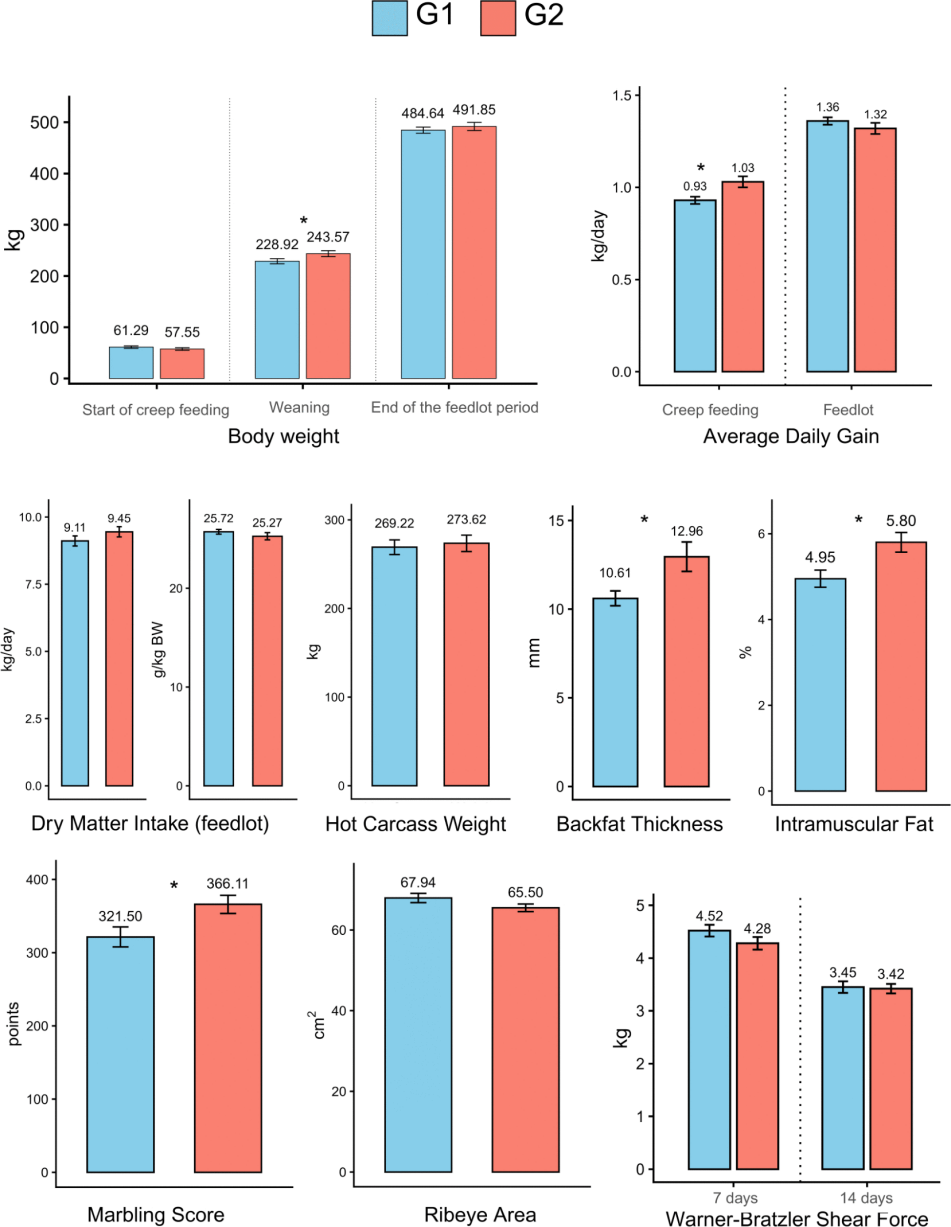
Performance, carcass, and meat quality traits of calves supplemented (G2) or not (G1) with creep-feeding. Values above bars represent means. Error bars indicate SEM. Asterisks (*) denote significant differences between treatments (*P* < 0.05).

Performance, carcass, and meat quality data for these animals (means and standard errors) were previously reported in Ramírez-Zamudio et al. [23] and are summarized in Fig 1. Groups did not differ in initial body weight at the start of the creep-feeding period (BWi: 61.29 ± 2.41 vs. 57.55 ± 2.61 kg for G1 and G2, respectively; *P* > 0.05). However, calves in the creep-feeding group (G2) had greater weaning weight (243.57 ± 5.70 vs. 228.92 ± 5.07 kg; *P* < 0.05) and higher pre-weaning average daily gain (ADG1: 1.03 ± 0.03 vs. 0.93 ± 0.02 kg/day; *P* < 0.05) than non-supplemented calves (G1). After weaning, there were no differences in final body weight (BWf: 491.85 ± 7.85 vs. 484.64 ± 5.96 kg), post-weaning average daily gain (ADG2: 1.32 ± 0.03 vs. 1.36 ± 0.02 kg/day), or hot carcass weight (HCW: 273.62 ± 9.28 vs. 269.22 ± 8.23 kg; *P* > 0.05). Dry matter intake during the feedlot period was also similar for both groups (P > 0.05), whether measured absolutely (9.45 ± 0.98 vs. 9.11 ± 1.28 kg/day) or adjusted for body weight (25.27 ± 3.01 vs. 25.72 ± 2.34 g/kg BW).

In terms of carcass and meat quality, creep-fed calves had significantly greater backfat thickness (BFT: 12.96 ± 0.83 vs. 10.61 ± 0.42 mm; *P* < 0.05), greater intramuscular fat percentage (IMF: 5.80 ± 0.23 vs. 4.95 ± 0.20%; *P* < 0.05), and higher marbling score (MS: 366.11 ± 12.39 vs. 321.50 ± 13.65 points; *P* < 0.05). Conversely, ribeye area (REA: 65.50 ± 0.93 vs. 67.94 ± 1.16 cm²) and Warner–Bratzler shear force after 7 and 14 days of aging (WBSF7: 4.28 ± 0.12 vs. 4.52 ± 0.11 kg; WBSF14: 3.42 ± 0.09 vs. 3.45 ± 0.11 kg) did not differ between treatments (*P* > 0.05).

Variables that differed significantly between groups (Weaning weight, ADG1, BFT, IMF, MS) showed moderate to high effect size estimates (Cohen’s d) ranging from 0.6 and 0.9 (S1 Table). In G1 (no creep) and G2 (creep-feeding) were equivalent (S2 Table), indicating low within-group variability in the random subsampling process.

### 3.2 RNA sequencing and mapping of reads to the reference genome

The mean concentration of total RNA obtained for the 24 samples was 260.27 ng/µL. The 260/280 and 260/230 nm ratios were approximately 1.9. The mean contamination with genomic DNA was 1.04% (range: 0.73% to 1.11%). The RIN was 7.6, ranging from 7.0 to 8.0. The mean contamination with genomic DNA was 1.04% (range: 0.73% to 1.11%). These results confirm that all samples had adequate integrity (RIN ≥ 7), were free of contaminants, and were therefore suitable for sequencing.

A total of 241.2 million PE reads (2 × 100 bp) were generated; of these, 230.5 million were uniquely mapped to the reference genome (uniquely mapped PE reads). Sequencing achieved a total depth of 37X, considering all transcripts from all samples. On average, 9.6 million uniquely mapped PE reads were obtained per sample, corresponding to 95.56% of the total PE reads generated.

The boxplots of the read counts normalized by the size factor showed that the distribution of quartiles was consistent between the samples of the two groups, indicating good quality of the sequencing data (S1 Fig). In relation to the expression profile of the constitutive genes, the expression was similar between the experimental groups (S2 Fig). PCA showed that the first two principal components explained more than 20% of the variation among samples (S3 Fig). In addition, the formation of clearly distinct groups of samples was observed at weaning, indicating an evident difference in the expression of genes between treatments.

The reads were mapped to a total of 27,607 genes, including protein-coding and non-protein-coding genes. To ensure the exclusive inclusion of genes with minimal relevant expression, a filtering criterion was applied that considered only those with uniquely mapped PE reads ≥ 3 in at least 12 samples. After filtering, the total number of expressed genes used in the count matrix for co-expression analysis was reduced to 16,604.

### 3.3 Co-expression modules

A total of 631 genes were grouped into seven modules (M1 to M6, and “not correlated”), organized in decreasing order of the number of genes per module (216, 145, 76, 70, 67, 44 and 13 genes, respectively) (S3 Table). The module with 13 genes was deemed “not correlated” due to the absence of significant enrichment (P-value adjusted > 0.05 in both groups) relative to the applied treatments. In this study, focus was directed towards Modules 1, 2, and 3 as they demonstrated the strongest associations and the highest absolute Normalized Enrichment Score (NES) values (S4 Table), obtained by GSEA. The NES indicates the predominance of a specific set of genes at the upper or lower end of a ranked list. In the case of this predominance, the gene set is considered to be enriched, while the random distribution of genes along the list indicates the absence of enrichment.

**Fig 2:**
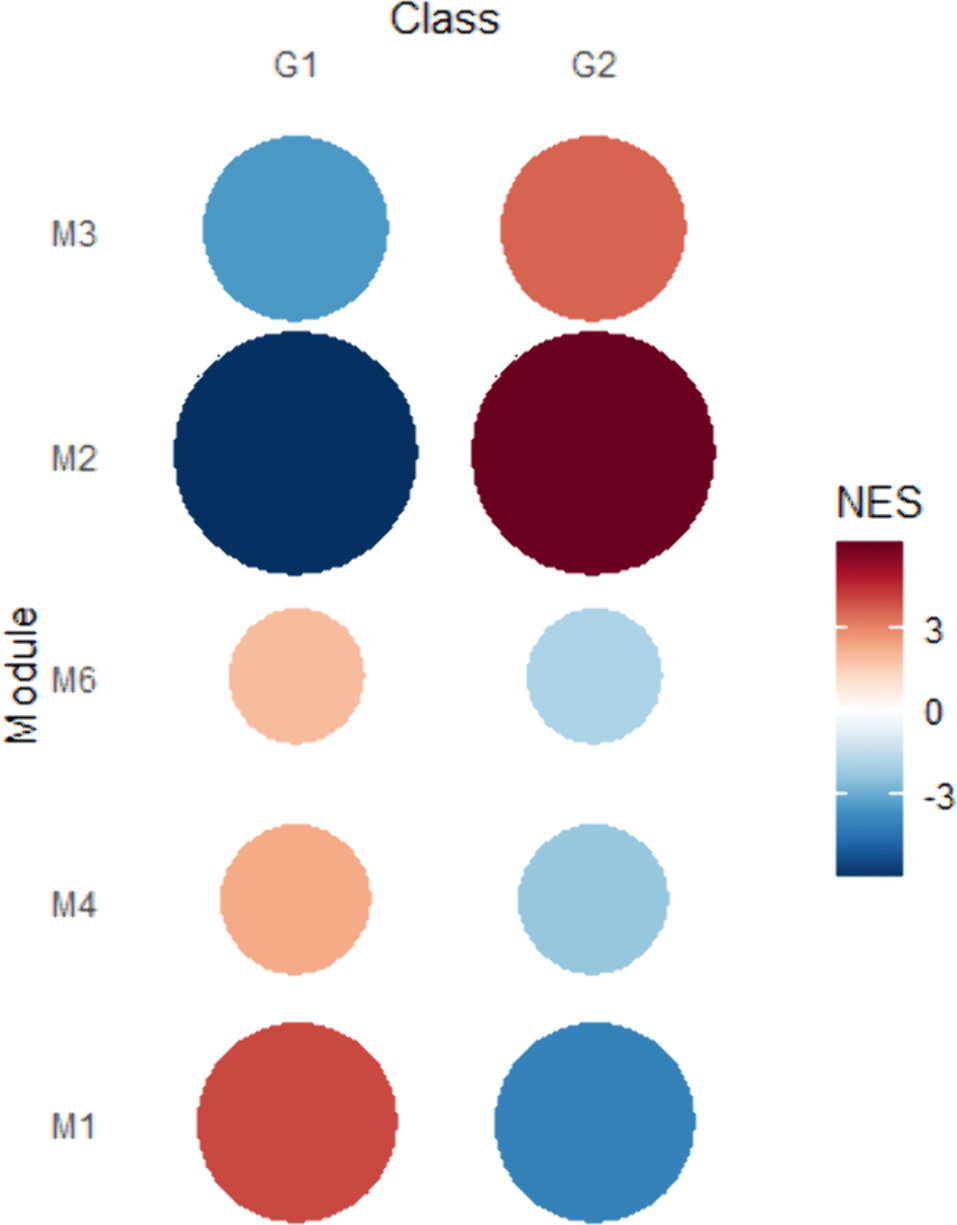
Gene set enrichment analysis. Heatmap of normalized enrichment scores (NES), where color and circumference indicate the score of each module in each experimental group (G1: no creep-feeding, G2: creep-feeding).

Among the three main modules, modules 2 and 3 showed the highest activity in G2, with an NES of 6.11 and 3.53, respectively, while the values were negative in G1 (NES = − 6.23 and – 3.53, respectively). In contrast, module 1 exhibited the opposite pattern, with the highest activity in G1 (NES = 4.08) and the lowest in G2 (NES = − 4.06). These results indicate that Module 2 and Module 3 were highly active in G2, while Module 1 exhibited the highest activity in G1, according to Fig 2 and S4 Table.

**Fig 3:**
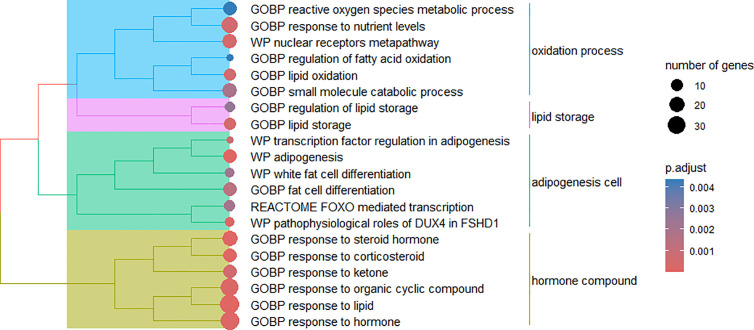
Cluster of biological mechanisms in module 1. Biological processes (GOBP) and WikiPathways (WP) and Reactome pathways enriched for genes present in module 1.

For module 1, overrepresentation analysis identified metabolic pathways and biological processes related to lipid catabolism and oxidation (Fig 3). These mechanisms, as well as the involved genes, help to explain the lower WW and lower subcutaneous and intramuscular fat deposition observed in G1 at slaughter. The main enriched metabolic pathways and biological processes were response to corticosteroid (GO:0031960), lipid oxidation (GO:0034440), response to ketone (GO:1901654), adipogenesis (WP987), nuclear receptors meta-pathway (WP2882), and FOXO-mediated transcription (bta9614085).

**Fig 4:**
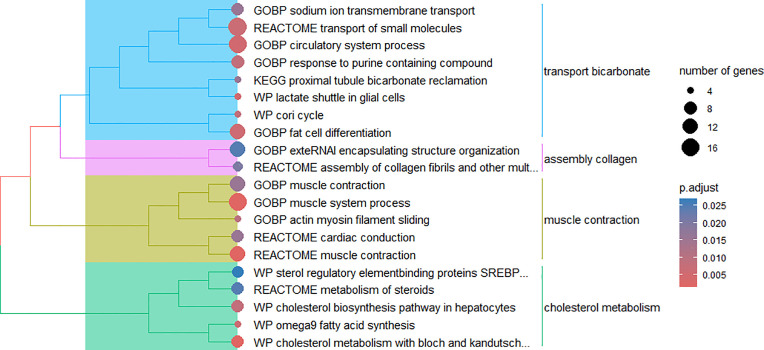
Cluster of biological mechanisms in module 2. Biological processes (GOBP) and WikiPathways (WP) and Reactome and Kyoto Encyclopedia of Genes and Genomes (KEGG) pathways enriched for genes present in module 2.

Several enriched metabolic pathways and biological processes were identified in module 2, which are related to the development and activity of important components such as muscle, adipose tissue, and the circulatory system (Fig 4). The enriched biological mechanisms include muscle system process (GO:0003012), muscle contraction (bta397014), fat cell differentiation (GO:0045444), omega-9 fatty acid synthesis (WP4724), cholesterol metabolism via Bloch and Kandutsch-Russell pathways (WP4718), and circulatory system process (GO:0003013).

**Fig 5:**
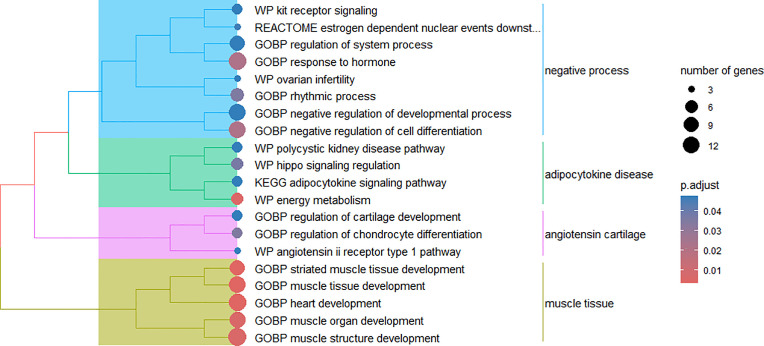
Cluster of biological mechanisms in module 3. Biological processes (GOBP) and WikiPathways (WP) and Reactome and Kyoto Encyclopedia of Genes and Genomes (KEGG) pathways enriched for genes present in module 3.

Enrichment of different biological processes related to muscle development, particularly striated muscle tissue development (GO:0014706) and muscle structure development (GO:0061061), is observed in module 3 and illustrated in Fig 5.

In general, modules 2 and 3 contain the biological mechanisms that can explain the higher WW and greater subcutaneous and intramuscular fat deposition in the group supplemented during the pre-weaning phase. All metabolic pathways and biological processes enriched for genes of modules 1, 2 and 3 are presented in S5-S7 Tables, respectively.

**Fig 6:**
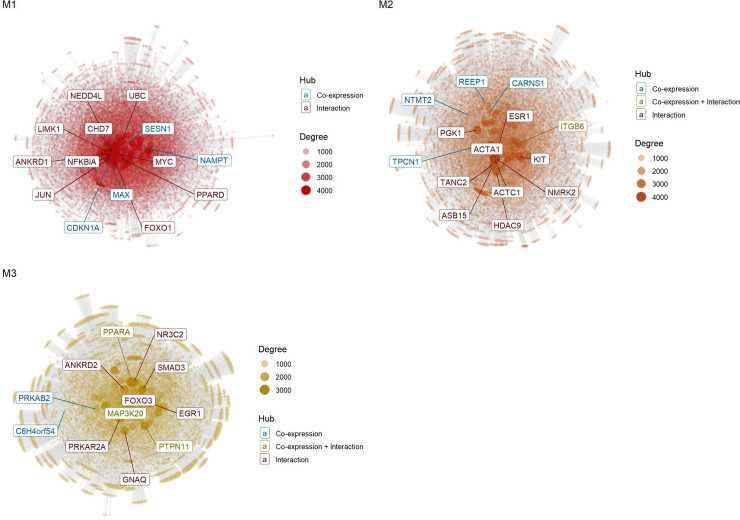
Gene interaction networks of modules, highlighting hub genes. Module 1(M1), module 2(M2), and module 3(M3).

Although hub genes were highlighted by interaction network analyses (Fig 6), the number of genes identified was still relatively high, a fact that makes it difficult to prioritize the most relevant genes. In these networks, the size of the nodes represents the degree of connection of each gene, highlighting those with the largest number of interactions. The colors of the boxes indicate the origin of the interactions: blue, when the interaction stems from co-expression analysis; red, when the interaction is derived exclusively from the inserted interactions file, and green, when there is an overlap between the two sources.

**Fig 7:**
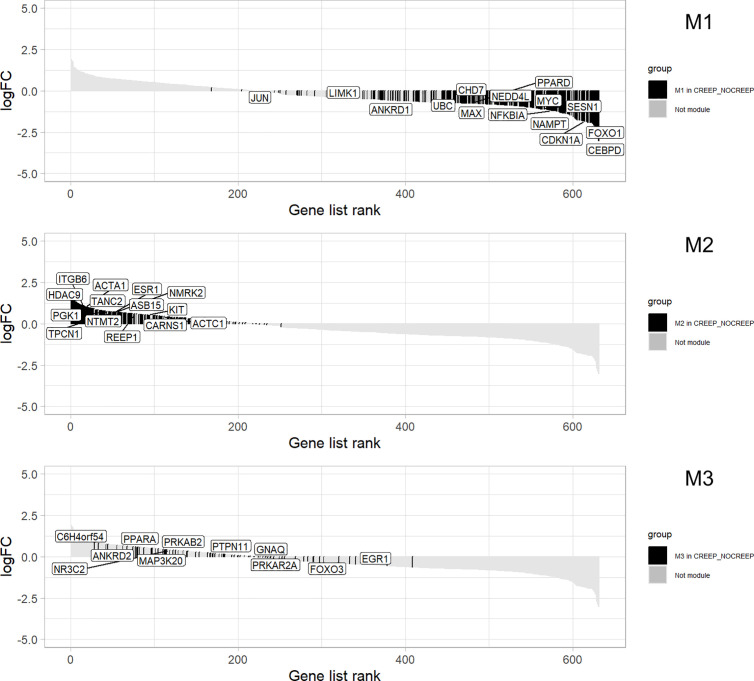
Distribution of hub genes in the GSEA ranked gene list in modules. Module 1(a), module 2(b), and module 3(c).

To prioritize the most relevant hub genes, we analyzed their distribution in the ranked list of genes generated by GSEA (Fig 7). This approach enabled the identification of genes that, in addition to acting as hubs, were also positioned at the ends of the ranked list. The main hub genes were *CDKN1A* (Cyclin Dependent Kinase Inhibitor 1A), *FOXO1* (Forkhead Box O1) and *NAMPT* (Nicotinamide Phosphoribosyltransferase) in module 1, mainly associated with metabolic adaptation to nutrient restriction and inhibition of adipogenesis; *ITGB6* (Integrin Subunit Beta 6), REEP1 (Receptor Accessory Protein 1) and *TPCN1* (Two Pore Segment Channel 1) in module 2, linked to lipid metabolism, adipogenesis and muscle differentiation; and *PPARA* (Peroxisome Proliferator Activated Receptor Alpha), *MAP3K20* (Mitogen-Activated Protein Kinase Kinase Kinase 20), and *PTPN11* (Protein Tyrosine Phosphatase Non-Receptor Type 11) in module 3, involved in energy metabolism and regulatory pathways of postnatal muscle growth and hypertrophy.

## 4 Discussion

### 4.1 Nutritional challenges and effects of creep-feeding in tropical systems

Supplementation is essential in tropical forage-based cattle farming systems, especially during the dry season when the nutritional quality of forages is markedly reduced [49,50]. This challenge is aggravated by the reduction in milk production of Nellore cows, which becomes insufficient to meet the nutritional requirements of calves by the third month of lactation, when the nutrient demand for growth is high [51]. This period can occur even earlier in F1 Angus × Nellore calves. As a consequence, obtaining nutrients becomes increasingly dependent on available forage, a fact that reinforces the importance of the strategic use of supplementation [49].

Creep-feeding during lactation can compensate for milk and pasture deficiencies, thereby stimulating growth, favoring fat deposition, and improving carcass finishing [52]. Results published previously for the calves used in the present study demonstrated an additional ADG of approximately 100 g and a 14.6 kg higher WW in animals that received supplement corresponding to 1% of body weight compared to unsupplemented animals [23].

After weaning, creep-feeding did not influence performance or traits such as REA and HCW. The lack of effect on final performance and carcass muscling (REA) may be related to the composition of gain [53,54], in which early lean tissue deposition reduces the potential for subsequent muscle growth, favoring fat deposition as the predominant component under high-energy diets [55]. Additionally, higher intramuscular fat (%F) content and marbling score (MS) were observed at slaughter, approximately 0.85% and 45 points greater in supplemented animals, respectively (Fig. 1), evidencing alterations in carcass composition. This increase in adipogenesis results from stimuli during a critical window, known as the marbling window, which extends from late prenatal development to approximately 250 days of age [56]. Additionally, higher intramuscular fat (%F) content and marbling score (MS) were observed at slaughter, approximately 0.85% and 45 points greater in supplemented animals, respectively (Fig. 1), showing changes in carcass composition. This increase in adipogenesis results from stimuli during a critical window, known as the marbling window, which spans from late prenatal development to approximately 250 days of age [56]. During this period, pre-weaning supplementation can increase the number of pre-adipocytes in skeletal muscle, favoring lipid biosynthesis during finishing [57]. It also represents a phase of metabolic imprinting, with long-lasting effects on the potential for intramuscular fat deposition throughout the animal’s productive life [58]. This aspect is reinforced by the findings of the present study, which, although not directly demonstrating early intramuscular fat formation, suggest that without this initial programming, fat deposition potential is limited, even with finishing high-energy diets. The transcriptional response observed at weaning, involving coexpression of genes from modules 2 and 3, including *PPARA*, *MAP3K20* and *PTPN11* (module 3) and *ITGB6*, *REEP1* and *TPCN1* (module 2), may represent an immediate adaptation to the nutritional stimulus during the suckling phase. This response may be associated with the superior performance of supplemented calves at weaning and the greater intramuscular fat content observed at slaughter (Fig 1). These molecular interactions likely reflect coordinated regulatory mechanisms linking early metabolic programming, muscle development and lipid accretion, which will be discussed in the following section.

### 4.2 Molecular responses to supplementation: regulatory pathways and key genes in modules 2 and 3

Postnatal growth is characterized by hypertrophy, mediated by the fusion of satellite cells to preexisting muscle fibers, which promotes an increase in fiber diameter [59] and in protein synthesis [60]. Energy demand is considerably increased during periods of accelerated growth, such as the prepubertal phase, in order to support protein deposition [61]. Within this context, supplementation during lactation is associated with increased metabolic activity in skeletal muscle, a tissue characterized by high protein turnover [62]. This adaptation is consistent with the co-expression of the *PPARA* gene observed in module 3, associated with the energy metabolism pathway (WP1541). The expression of *PPARA* in skeletal muscle is linked to the activation of oxidation for energy production [63], which is consistent with the high energy requirements of protein synthesis at this stage of growth.

In addition to the energy demand associated with growth, the higher expression of *PPARA* can also be attributed to diet composition since the supplement provided to the animals consisted of 40.4% soybean meal, an ingredient rich in unsaturated fatty acids [64]. Although ruminal biohydrogenation partially alters the lipid profile, a proportion of these fatty acids can reach the skeletal muscle [23], promoting the activation of *PPARA*. Indeed, [64] demonstrated that soy-based diets increase the concentration of unsaturated fatty acids such as oleic and linolenic acids in muscle, which are positively correlated with the expression of *PPARA*.

Skeletal muscle is composed of a heterogeneous group of cells, including muscle fibers, adipocytes, and fibroblasts [56]. Among these cells, *PPARA* is mainly expressed in muscle fibers [65], reinforcing its role as a metabolic sensor involved in the regulation of energy metabolism [66]. In this context, mechanisms associated with higher energy demand may have been activated to support hypertrophic activity via satellite cells in supplemented calves, as suggested by the upregulation of *TPCN1* (module 2), as well as *PTPN11* and *MAP3K20* (module 3). These genes were co-expressed under the conditions studied and are associated with muscle growth and muscle structure processes, reflected in the enrichment of muscle contraction (bta397014) and muscle system process (GO:0003012) pathways in module 2, in addition to muscle tissue development (GO:0060537), striated muscle tissue development (GO:0014706), and muscle structure development (GO:0061061) in module 3.

The activation of satellite cells during skeletal muscle hypertrophy follows a widely accepted model, in which the expression of transcription factors such as Pax3, Pax7 and Myf5 is essential for the proliferation of these cells [67,68]. Subsequently, MyoD [69] and MyoG are activated, promoting the differentiation and fusion of satellite cells with pre-existing muscle fibers. As satellite cells shift from the proliferative state to differentiation and subsequent fusion into muscle fibers, the expression of key myogenic regulators is largely suppressed, with the exception of MyoG, which remains active and is essential for terminal differentiation [70].

In the present study, the *PTPN11* gene, which encodes protein SHP2 [71], can be highlighted because of its role in regulating the proliferation of myogenic cells during postnatal life, as described by [72]. *MAP3K20*, a member of the MAPKKK family of signal transduction proteins, participates positively in myogenic differentiation through the regulation of the JNK/MAPK pathway [73] and has also been identified as a key hub gene in bovine muscle development [74]. The *TPCN1* gene, which belongs to the family of endolysosome-targeted calcium release channels, is involved in cell differentiation, including skeletal muscle, through the NAADP-mediated release of calcium [75,76]. The co-expression of these three genes observed in the present study reinforces the role of creep-feeding supplementation in the stimulation of molecular mechanisms associated with postnatal myogenesis and muscle hypertrophy. These mechanisms may be related to the greater daily weight gain and WW previously observed by [23] in supplemented calves compared to G1.

In addition to these mechanisms of muscle hypertrophy, attention should also be given to the period known as the marbling window, which extends to approximately 250 days of life in cattle [56,57]. This stage represents a strategic period during which early nutritional interventions can induce metabolic imprinting and favor long-term intramuscular fat deposition [77]. In this context, *ITGB6*, a hub gene identified in module 2, plays an important role in adipogenesis [78,79] and angiogenesis [80]. These processes are closely related to the development and remodeling of adipose tissue [81]. The product encoded by the *ITGB6* gene belongs to the integrin family and acts as a mediator of cell-extracellular matrix signaling, playing a key role in tissue remodeling [82]. Its activation has also been associated with angiogenesis in physiological and pathological contexts [80]. The enrichment of fat cell differentiation (GO:0045444) and circulatory system process (GO:0003013) observed in this study reinforces the role of *ITGB6* in coordinating adipogenesis and angiogenesis, which act in an integrated manner in adipose tissue formation and functionality. Angiogenesis is essential for the expansion of adipose tissue, ensuring oxygenation, nutrient supply, and the transport of hormones and growth factors that regulate metabolism and remodeling of this tissue [83,84]. This molecular signature suggests that *ITGB6* acts as a central mediator linking extracellular matrix remodeling and vascular signaling, thereby defining an adipogenic niche. Because adipose tissue is highly vascularized and adipogenesis is spatially and temporally coupled with angiogenesis [85], this coordination may partly explain the greater intramuscular fat deposition observed in the supplemented group.

Building on these processes, the activation of angiogenic pathways during the weaning period may indicate a mechanism preparing the microenvironment for more efficient lipogenesis during the finishing phase. The enrichment of cholesterol metabolism via the Bloch and Kandutsch-Russell pathways (WP4718) and omega-9 fatty acid synthesis (WP4724) is consistent with the view that the adipose tissue of supplemented calves was metabolically activated to support lipogenesis. Cholesterol is essential for membrane formation and can modulate lipogenic signaling [86]. Furthermore, cholesterol metabolism is related to the expression of the *SCD1* gene [87], which participates in the first steps of lipogenesis, particularly in fatty acid desaturation [88].

In the study by [23], *SCD1* was found to be upregulated in supplemented calves, although it was not detected in the present dataset. However, the co-expression of *REEP1*, identified as a hub gene in module 2, may suggest a functional connection with *SCD1*. *REEP1* plays a role in lipid metabolism, mediated by specific interactions with proteins such as seipin and atlastin-1, which directly regulate lipid homeostasis [89]. Seipin, in turn, is distributed throughout the endoplasmic reticulum and interacts with various proteins involved in lipid droplet biogenesis and lipogenesis, particularly *SCD1* [90]. Seipin has been proposed to act as a regulatory complex, integrating key components of lipid metabolism and controlling the trafficking of phospholipids to lipid droplets [91,92]. Thus, although *SCD1* was not directly detected in the present study, the presence of *REEP1* as a hub gene, along with the activation of WP4718 and WP4724 pathways, is consistent with the induction of lipogenic mechanisms. Among these processes, the formation of monounsaturated fatty acids such as oleic acid should be highlighted. The biosynthesis of these fatty acids in growing cattle is attributed to the enzyme Δ9-desaturase, encoded by the *SCD* gene [93]. This metabolic activation may contribute to explain the greater intramuscular fat deposition in supplemented animals reported by [23], supporting the possibility that such mechanisms were triggered early in life.

### 4.3 Metabolic adjustments and gene expression in skeletal muscle of unsupplemented calves: regulatory pathways and key genes in module 1

In tropical systems, calves born to Zebu cows face increasing nutritional challenges during the lactation period since milk production becomes insufficient to fully meet their requirements [51]. As lactation progresses, these animals become dependent on forage, whose nutritional value declines due to seasonality [49,50]. The difference in WW of 14.6 kg observed by [23] in G1 calves is consistent with the interpretation that, even while growing, these animals faced progressive nutritional limitations during lactation, which may have led to required metabolic adjustments in skeletal muscle. In the present study, the enrichment of biological processes such as response to corticosteroids (GO:0031960), lipid oxidation (GO:0034440), and response to ketone (GO:1901654) is consistent with an increase in the mobilization of alternative energy substrates. Furthermore, enrichment of the adipogenesis (WP987), nuclear receptors meta-pathway (WP2882), and FOXO-mediated transcription (bta9614085) pathways is inline with regulatory mechanism associated with metabolic adaptation in response to lower nutrient availability, even under conditions of growth.

The hub genes *FOXO1*, *CDKN1A*, and *NAMPT* were identified in module 1. These genes are negatively associated with the development of adipose tissue [94–97]. Regulation of *FOXO1*, a transcription factor of the Forkhead family, plays a relevant role in the modulation of insulin-mediated glucose metabolism, fatty acid oxidation, and adipogenesis through the recruitment of preadipocytes [98,99]. *FOXO1* exhibits a dual role in adipogenesis: during the early stages of adipocyte differentiation, it promotes adipogenesis [98,99], whereas in post-mitotic stages, *FOXO1* inhibits this process by regulating *CDKN1A*, which encodes protein p21, an inhibitor of the transition from the G1 to the S phase of the cell cycle [94,95]. In addition, one of FOXO1’s inhibitory functions in adipogenesis involves suppressing the master transcription factor PPARγ, which is essential during the final stages of adipocyte differentiation [100]. Under different nutritional states, *FOXO1* inactivation or activation, mediated by insulin and glucagon signaling, respectively, promotes lipogenic or lipid-catabolic molecular programs [98,101,102]. This is consistent with the stronger association of *FOXO1* with the module correlated with non-complemented calves during the suckling phase. In this context, the relatively higher expression of *FOXO1* during the marbling period aligns with limited pre-adipocyte differentiation, resulting in fewer adipocytes available for lipid deposition during the finishing phase. This interpretation is supported by [23] who, using the same animals as studied here, reported a reduction of 0.85% in the intramuscular fat content of meat in G1. According to [64], at the end of the marbling window, the effects of nutritional interventions are restricted to hypertrophy of already formed adipocytes. This fact limits the potential of increasing MS, even when finishing diets with a high proportion of concentrate are administered.

On the other hand, expression of the *NAMPT* gene in unsupplemented calves is consistent with an adaptative mechanism to environments with lower nutrient availability, given its activity in situations of high energy demand such as cell differentiation [97]. Under conditions of low nutrient supply, *NAMPT* expression is elevated in both skeletal muscle [103] and subcutaneous adipose tissue [104]. This activation may reflect a functional associated with the expression of *FOXO1* and *CDKN1A* (Fig. 6 and 7), which together appear to limit adipocyte proliferation while prioritizing energy utilization for processes that are more critical for development, such as protein synthesis in skeletal muscle. This interpretation is consistent with the growth stage of calves before puberty, a period that requires considerable energy supply for muscle hypertrophy [61].

### 4.4 Study limitations

Some limitations of this study should be noted. The number of animals used for transcriptome analysis (n = 12 per group) aligns with the common practice in RNA-Seq experiments with cattle. However, this sample size reduces the sensitivity to detect smaller transcriptional differences. The fatty acid composition of intramuscular fat was not determined, which limits detailed exploration of lipid metabolism. Both groups were managed with the same finishing diet, so differences in fatty acid profiles were not expected. The variation in intramuscular fat observed is thus more likely linked to early nutritional modulation during the pre-weaning period. Co-expression analyses using CEMiTool identify coordinated gene activity but do not demonstrate causality. Further functional studies are needed to confirm the specific roles of the hub genes highlighted here.

## 5 Conclusion

Creep-feeding supplementation during lactation was associated with lasting changes in skeletal muscle gene expression of F1 Angus × Nellore calves, reflected in pathways related to myogenesis, adipogenesis, angiogenesis, and energy metabolism, thereby creating a favorable molecular environment for muscle growth and intramuscular fat deposition, consistent with the higher weaning weight and improved carcass finishing observed in supplemented animals. In contrast, the absence of supplementation was linked to adaptive responses to nutrient restriction, with enrichment of processes related to lipid oxidation, metabolic stress, and inhibition of myogenesis and adipogenesis. These findings suggest that early nutritional interventions applied during the “marbling window” may favor intramuscular fat deposition in Angus × Nellore crossbred calves raised under tropical conditions, where pasture seasonality combined with the decline in milk production of cows limits nutrient availability for calves. Under the conditions of this study, the results indicate that creep-feeding supplementation during the pre-weaning phase is a practical management strategy with the potential to improve meat quality at finishing.

## Supporting information

S1 Fig Log2 boxplot of the read count normalized by the size factor per sample in G1 (control, no creep-feeding) and G2 (creep-feeding). (DOCX)

S2 Fig Expression profile of reference genes in the control group (G1, no creep-feeding) and the group submitted to creep-feeding (G2). (DOCX)

S3 Fig Principal component analysis (PCA) performed based on normalized count data of gene expression for samples collected at weaning from G1 (control, no creep-feeding) and G2 (creep-feeding). (DOCX)

S1 Table Means, standard deviations (SD), and effect size estimates (Cohen’s d) for growth performance, carcass, and meat quality traits in control (G1, no creep) and creep-feeding (G2) F1 Angus x Nellore calves. (DOCX)

S2 Table Equivalence testing (Two One-Sided Tests; TOST) for growth performance, carcass, and meat quality traits in F1 Angus x Nellore calves from control (no-creep) and creep-feeding groups. (DOCX)

S3 Table Identification and quantification of the genes in each of the modules generated by CEMITool. (DOCX)

S4 Table NES values and adjusted p-values generated by GSEA for each module in each group. G1 = Group 1 (without creep-feeding), G2 = Group 2 (with creep-feeding), NES = Normalized Enrichment Score, GSEA = Gene Set Enrichment Analysis, Adjusted p-value for FDR (False Discovery Rate).
(DOCX)

S5 Table All terms from module 1 (GO Biological Process, KEGG pathways, REACTOME pathways, and WikiPathways) with adjusted p-value < 0.05. (DOCX)

S6 Table All terms from module 2 (GO Biological Process, KEGG pathways, REACTOME pathways, and WikiPathways) with adjusted p-value < 0.05. (DOCX)

S7 Table All terms from module 3 (GO Biological Process, KEGG pathways, REACTOME pathways, and WikiPathways) with adjusted p-value < 0.05. (DOCX)
