# Supplementary material for: Effect of creep-feeding supplementation during the pre-weaning phase on gene co-expression in Longissimus thoracis muscle of F1 Angus x Nellore calves at weaning
Source: PLoS One. 2025 Dec 18;20(12):e0339043. doi: 10.1371/journal.pone.0339043 (PMC12714228; doi:10.1371/journal.pone.0339043)
Supplement: S1 Fig — (DOCX) [file pone.0339043.s001.docx]

**
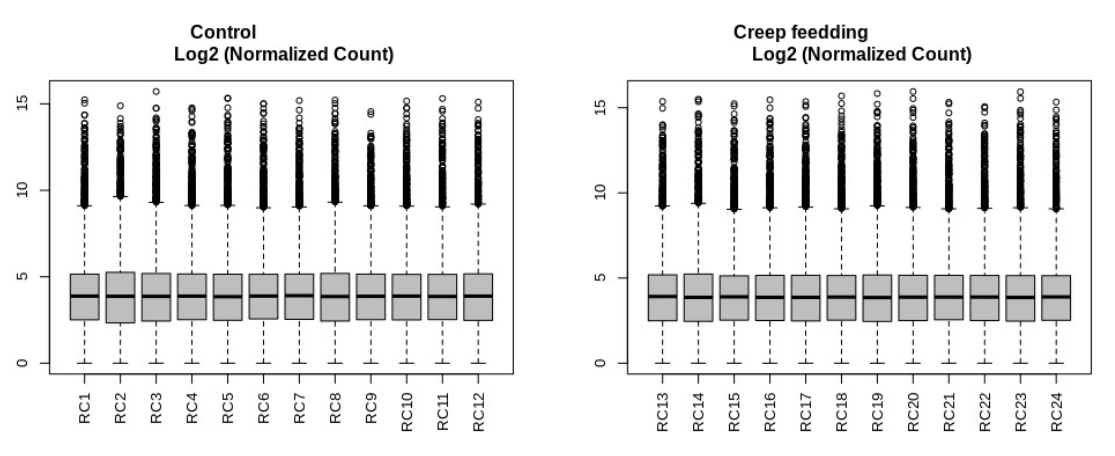
S1 Figure. Log2 boxplot of the read count normalized by the size factor per sample in G1 (control, no creep-feeding) and G2 (creep-feeding).**
