# Supplementary material for: Effect of creep-feeding supplementation during the pre-weaning phase on gene co-expression in Longissimus thoracis muscle of F1 Angus x Nellore calves at weaning
Source: PLoS One. 2025 Dec 18;20(12):e0339043. doi: 10.1371/journal.pone.0339043 (PMC12714228; doi:10.1371/journal.pone.0339043)
Supplement: S2 Fig — (DOCX) [file pone.0339043.s002.docx]

**
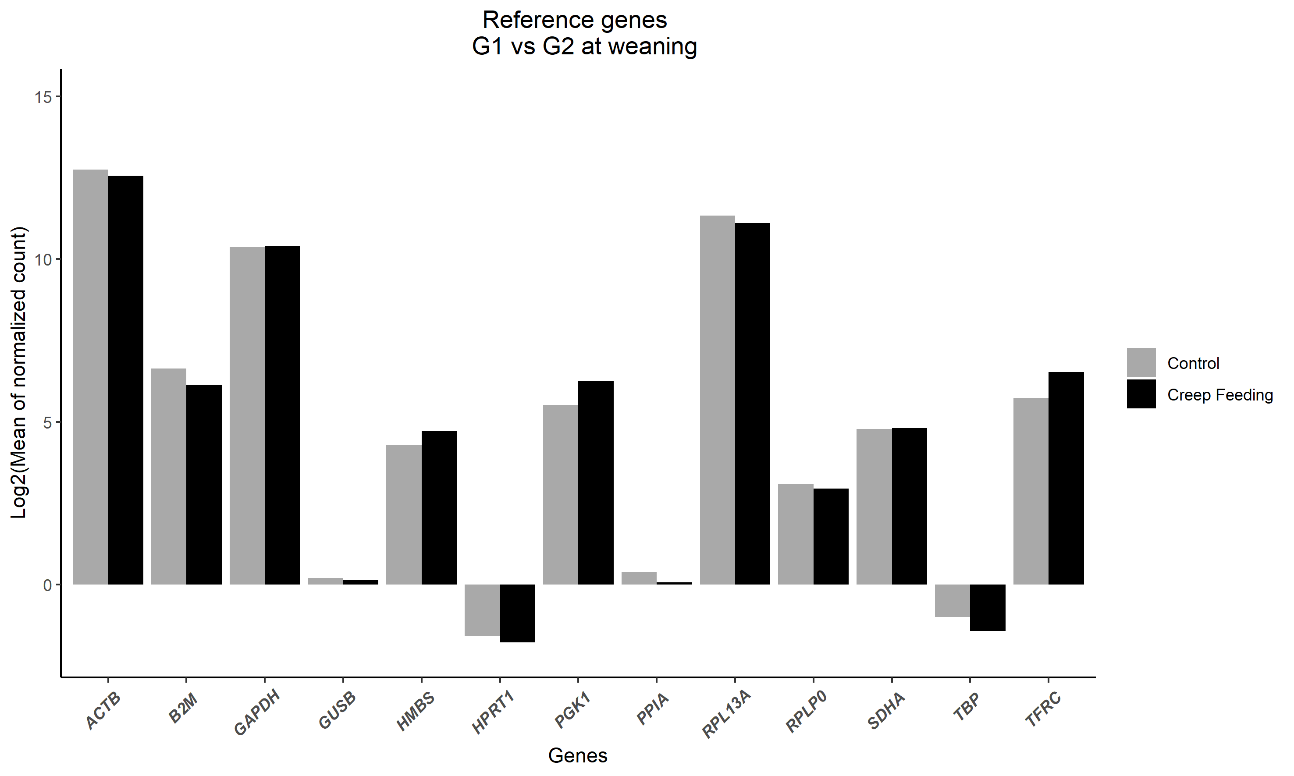
S2 Figure. Expression profile of reference genes in the control group (G1, no creep-feeding) and the group submitted to creep-feeding (G2).**
