# Supplementary material for: Effect of creep-feeding supplementation during the pre-weaning phase on gene co-expression in Longissimus thoracis muscle of F1 Angus x Nellore calves at weaning
Source: PLoS One. 2025 Dec 18;20(12):e0339043. doi: 10.1371/journal.pone.0339043 (PMC12714228; doi:10.1371/journal.pone.0339043)

**S3 Figure. Principal component analysis (PCA) performed based on normalized count data of gene expression for samples collected at weaning from G1 (control, no creep-feeding) and G2 (creep-feeding).**


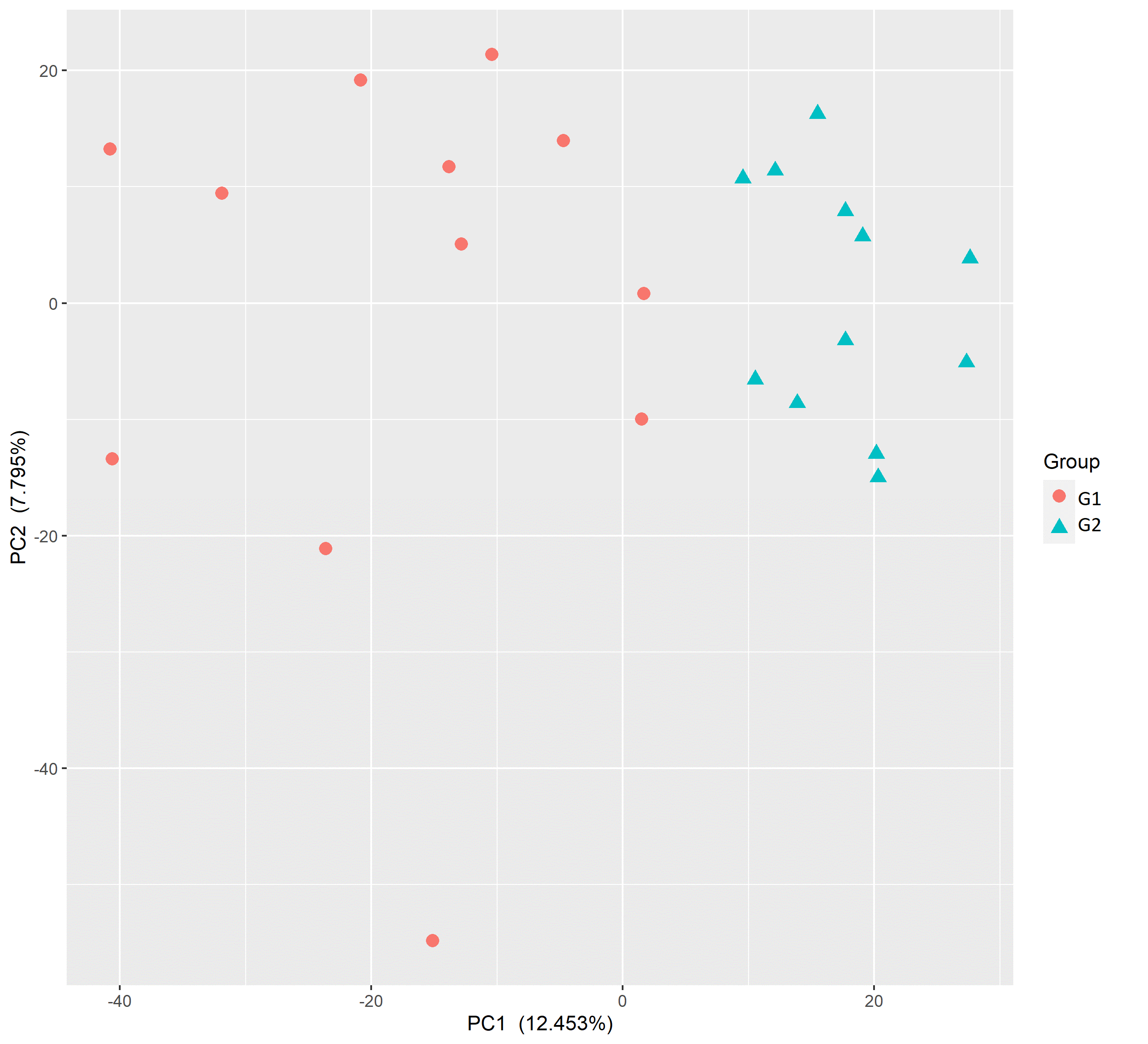

Supplement: S3 Fig — (DOCX) [file pone.0339043.s003.docx]
