# Supplementary material for: Effect of creep-feeding supplementation during the pre-weaning phase on gene co-expression in Longissimus thoracis muscle of F1 Angus x Nellore calves at weaning
Source: PLoS One. 2025 Dec 18;20(12):e0339043. doi: 10.1371/journal.pone.0339043 (PMC12714228; doi:10.1371/journal.pone.0339043)
Supplement: S1 Table — (DOCX) [file pone.0339043.s004.docx]

| **Trait** | **Unit** | **G1 mean** | **G1 SD** | **G2 mean** | **G2 SD** | **Cohen’s *d*** |
| --- | --- | --- | --- | --- | --- | --- |
| Initial body weight (BWi) | kg | 61.29 | 11.80654 | 57.55 | 12.78634 | -0.304 |
| Weaning weight | kg | 228.92 | 24.83783 | 243.57 | 27.92418 | 0.554 |
| Pre-weaning ADG (ADG1) | kg/day | 0.93 | 0.09798 | 1.03 | 0.146969 | 0.801 |
| Final body weight (BWf) | kg | 484.64 | 29.19792 | 491.85 | 38.45699 | 0.211 |
| Post-weaning ADG (ADG2) | kg/day | 1.36 | 0.09798 | 1.32 | 0.146969 | -0.32 |
| Hot carcass weight (HCW) | kg | 269.22 | 40.3186 | 273.62 | 45.46253 | 0.102 |
| Dry matter intake (absolute) | kg/day | 9.11 | 6.270694 | 9.45 | 4.801 | 0.061 |
| Dry matter intake (g/kg BW) | g/kg BW | 25.72 | 11.46361 | 25.27 | 14.74593 | -0.034 |
| Backfat thickness (BFT) | mm | 10.61 | 2.057571 | 12.96 | 4.066153 | 0.729 |
| Intramuscular fat (IMF) | % | 4.95 | 0.979796 | 5.8 | 1.126765 | 0.805 |
| Marbling score (MS) | points | 321.5 | 66.87107 | 366.11 | 60.69836 | 0.699 |
| Ribeye area (REA) | cm² | 67.94 | 5.682816 | 65.5 | 4.556051 | -0.474 |
| Shear force after 7 days (WBSF7) | kg | 4.52 | 0.538888 | 4.28 | 0.587878 | -0.426 |
| Shear force after 14 days (WBSF14) | kg | 3.45 | 0.538888 | 3.42 | 0.440908 | -0.061 |

**S1 Table. Means, standard deviations (SD), and effect size estimates (Cohen’s d) for growth performance, carcass, and meat quality traits in control (G1, no creep) and creep-feeding (G2) F1 Angus x Nellore calves.**
