# Supplementary material for: Effect of creep-feeding supplementation during the pre-weaning phase on gene co-expression in Longissimus thoracis muscle of F1 Angus x Nellore calves at weaning
Source: PLoS One. 2025 Dec 18;20(12):e0339043. doi: 10.1371/journal.pone.0339043 (PMC12714228; doi:10.1371/journal.pone.0339043)
Supplement: S2 Table — (DOCX) [file pone.0339043.s005.docx]

**S2 Table.** **Equivalence testing (Two One-Sided Tests; TOST) for growth performance, carcass, and meat quality traits in F1 Angus x Nellore calves from control (no-creep) and creep-feeding groups.**

| **Group** | **Trait** | **Unit** | **Low eq bound** | **High eq bound** | **Difference** | ***P* eq** | **Decision** |  |
| --- | --- | --- | --- | --- | --- | --- | --- | --- |
| No-creep | Backfat thickness (BFT) | mm | -1.826 | 1.826 | 0.19 | 0.00784 | Equivalent |  |
| No-creep | Dry matter intake (absolute) | kg/day | -5.248 | 5.248 | -0.06 | 0.00374 | Equivalent |  |
| No-creep | Dry matter intake (g/kg BW) | g/kg BW | -10.062 | 10.062 | -0.32 | 0.00469 | Equivalent |  |
| No-creep | Final body weight (BWf) | kg | -26.204 | 26.204 | -2.14 | 0.00689 | Equivalent |  |
| No-creep | Hot carcass weight (HCW) | kg | -35.401 | 35.401 | -1.82 | 0.00538 | Equivalent |  |
| No-creep | Initial body weight (BWi) | kg | -10.753 | 10.753 | -0.49 | 0.00558 | Equivalent |  |
| No-creep | Intramuscular fat (IMF) | % | -0.854 | 0.854 | -0.07 | 0.00654 | Equivalent |  |
| No-creep | Marbling score (MS) | points | -56.125 | 56.125 | 2.8 | 0.00493 | Equivalent |  |
| No-creep | Post-weaning ADG (ADG2) | kg/day | -0.118 | 0.118 | -0.02 | 0.0218 | Equivalent |  |
| No-creep | Pre-weaning ADG (ADG1) | kg/day | -0.101 | 0.101 | 0.01 | 0.0105 | Equivalent |  |
| No-creep | Ribeye area (REA) | cm² | -5.006 | 5.006 | -0.14 | 0.0046 | Equivalent |  |
| No-creep | Shear force after 14 days (WBSF14) | kg | -0.443 | 0.443 | -0.02 | 0.00464 | Equivalent |  |
| No-creep | Shear force after 7 days (WBSF7) | kg | -0.495 | 0.495 | -0.02 | 0.00549 | Equivalent |  |
| No-creep | Weaning weight | kg | -21.772 | 21.772 | 0.58 | 0.00451 | Equivalent |  |
| Creep-feeding | Backfat thickness (BFT) | mm | -3.436 | 3.436 | -0.26 | 0.00596 | Equivalent |  |
| Creep-feeding | Dry matter intake (absolute) | kg/day | -4.029 | 4.029 | -0.07 | 0.00391 | Equivalent |  |
| Creep-feeding | Dry matter intake (g/kg BW) | g/kg BW | -12.916 | 12.916 | -0.17 | 0.0041 | Equivalent |  |
| Creep-feeding | Final body weight (BWf) | kg | -33.258 | 33.258 | -2.95 | 0.00676 | Equivalent |  |
| Creep-feeding | Hot carcass weight (HCW) | kg | -38.666 | 38.666 | -0.82 | 0.00411 | Equivalent |  |
| Creep-feeding | Initial body weight (BWi) | kg | -11.416 | 11.416 | 0.55 | 0.00544 | Equivalent |  |
| Creep-feeding | Intramuscular fat (IMF) | % | -1.031 | 1.031 | -0.04 | 0.00539 | Equivalent |  |
| Creep-feeding | Marbling score (MS) | points | -54.251 | 54.251 | -1.41 | 0.00468 | Equivalent |  |
| Creep-feeding | Post-weaning ADG (ADG2) | kg/day | -0.125 | 0.125 | -0.01 | 0.00622 | Equivalent |  |
| Creep-feeding | Pre-weaning ADG (ADG1) | kg/day | -0.143 | 0.143 | -0.01 | 0.00764 | Equivalent |  |
| Creep-feeding | Ribeye area (REA) | cm² | -4.097 | 4.097 | 0.2 | 0.00555 | Equivalent |  |
| Creep-feeding | Shear force after 14 days (WBSF14) | kg | -0.411 | 0.411 | -0.03 | 0.00706 | Equivalent |  |
| Creep-feeding | Shear force after 7 days (WBSF7) | kg | -0.554 | 0.554 | 0.01 | 0.00504 | Equivalent |  |
| Creep-feeding | Weaning weight | kg | -24.181 | 24.181 | -2.27 | 0.00702 | Equivalent |  |
|  |  |  |  |  |  |  |  |  |
