# Supplementary material for: Effect of creep-feeding supplementation during the pre-weaning phase on gene co-expression in Longissimus thoracis muscle of F1 Angus x Nellore calves at weaning
Source: PLoS One. 2025 Dec 18;20(12):e0339043. doi: 10.1371/journal.pone.0339043 (PMC12714228; doi:10.1371/journal.pone.0339043)
Supplement: S3 Table — (DOCX) [file pone.0339043.s006.docx]

**S3 Table. Identification and quantification of the genes in each of the modules generated by CEMITool.**

| Module | Genes | Number of genes |
| --- | --- | --- |
| M1 | *TXNIP, NRAP, ANKRD1, PPP1R27, GLUL, ZFAND5, PDK4, DDIT4, MUSTN1, PFKFB4, RILPL1, UNG, CYB5R1, SLC25A34, UBC, XDH, XIRP1, PSME4, PNRC1, JUN, SORBS1, KLHL40, STAT5B, TRIM63, UCP3, RBM38, MYH14, PNPLA2, FKBP5, ART3, KLF10, PER1, BNIP3, SCARF1, RAPSN, EIF4EBP1, MGLL, NAMPT, REV3L, RIOK3, FTL, NEDD4L, PFKFB3, RNF144B, SLC7A8, ZFP36, SESN1, MTHFR, LPIN1, NFKBIA, ARRDC2, GABARAPL1, FBXO32, TTC9, MAX, CCN1, RPS6KA3, ARID5B, STRIP2, TCIM, BCL2L1, CEBPB, HSF4, CTSF, ADIPOR2, ZBTB16, ACOX3, KLF9, CPT1B, KLF4, CHD7, GSTM3, ALAD, FNTB, TMEM120B, MASP1, CEBPD, DDO, THOP1, ASS1, HACL1, TINAGL1, SLC43A2, IL32, TMEM140, PRSS23, CISH, PPP1R15A, PPDPFL, SPOCK2, GADD45A, USP54, TNFRSF12A, ABCA1, RGCC, S100A13, KLF11, BTNL9, LOC521224, HOXA9, WDR91, PDE4B, EIF4E3, IL6R, LOC112444653, OSBPL11, ANKRD28, SIK1, MGC138914, SLC7A4, SLC26A10, FOXS1, UCP2, ADAM9, ISOC2, KLHL30, CDKN1A, PPARGC1A, THBS1, ASCC1, GADD45G, ENOX2, UROS, HABP2, MLYCD, LOC100300896, GCNT1, DHRS12, MAP1A, PDXK, PLIN5, TRIM45, NAPRT, EPDR1, SNHG12, FOXO1, KLF15, POLR2I, CRLF3, BANP, SIK2, PPARD, ANGPTL4, APOE, SDC4, GPX3, VNN1, HMOX1, CSRNP1, LOC618409, APOLD1, LOC104968411, LOC618664, ZNF703, ATOH8, NPC1, SPR, METRNL, TST, FOSL2, SH3RF2, LIMK1, SEMA3B, DNAJC1, C24H18orf21, PALS2, VOPP1, METTL21C, PTPRK, MT2A, CREB5, HTATIP2, TPRN, MOSPD1, LOC785216, GREB1L, MOB3B, CPT1A, AATF, RET, SCN3B, RIN1, WBP1L, PM20D2, ARMC12, IMPA2, CDC14A, TBX3, LOC507756, MYC, SOCS2, LOC515676, MAOA, AMPD3, SLC22A16, CNTFR, LIPE, PTPN3, MNT, GLCE, LGALS4, NMT2, CIART, RSPO3, EFHB, SMOC1, CLCF1, ALPL, ME3, ANGEL1, PLSCR4, DEPP1, CREM, FAH, KCNMB4, CBR3* | 216 |
| M2 | *ACTA1, MYL1, MYH2, CA3, LDHA, NMRK2, PGK1, AGL, MYL6B, MYL3, MYH7B, MSS51, CAMK2A, NREP, COL15A1, SEMA6C, SCN4B, PPP1R3C, LMCD1, SLC2A4, GADL1, AQP1, CST6, IGFBP5, CCDC69, SCD, LPL, BHLHE40, SLC16A3, ETNPPL, FABP3, SLC9A5, CASQ2, CD93, KCNC4, DNAJA4, CHRNE, REEP1, CA2, SLC12A2, ABRA, SLC6A1, ASB15, FASN, PPM1K, IGDCC4, FRZB, DHRS7B, CARNS1, CCDC80, KCTD15, GPT, ATP2B2, P2RY1, GRIP2, FADS2, VASH1, LOC100848815, IQCA1L, NPNT, CDC42EP3, P2RY2, DUSP10, ADAMTS20, ACTC1, ESR1, LRRN1, ITGB6, C1QTNF3, DHDH, GATM, SETBP1, LUM, KY, CCDC162P, CYB5R2, PLPPR2, LOC508916, DENND2A, SPX, ACTBL2, SAMD11, CACNA2D2, PMEPA1, CACNG4, LOC788915, EXTL1, AQP4, FMOD, PIGZ, TMEM51, MAFB, FADS1, ACKR3, TANC2, COL11A2, CARNMT1, INHBB, TPCN1, COL14A1, POPDC2, ATP1A4, KCP, DLK1, ADAMTS8, PCDH12, RFLNB, FSD1L, MIR1-2, NTMT2, LSS, TNFAIP8L1, SIX2, KIT, GPIHBP1, SLC2A12, COL27A1, CDC42EP2, INKA1, ZNF608, LAMA3, HDAC9, REEP2, SLC4A4, CHODL, ANGPT2, OLFML2B, ST8SIA2, FBXL22, ZNF385A, PLXDC1, HRH2, SNAI3, NPR3, NRK, TAGLN3, INSIG1, MYLK4, CCDC159, AGBL1, SRD5A1, CYP51A1, DHCR24, PALD1* | 145 |
| M3 | *XIRP2, ZNF106, CSRP3, HSPB7, TFRC, PLN, MAP3K20, C6H4orf54, ANKRD2, USP13, PRKAR2A, PADI2, AKAP6, PRKAB2, RETREG1, GAB2, ANO6, CLIC5, HK2, LDAF1, HLF, PTPN11, HBEGF, NT5C1A, HOOK3, NRIP1, DLL4, PRKAA2, SLC9A2, SOX6, LNPEP, PPARA, MITF, CCN2, PGM2L1, GYG2, TMEM245, SORT1, FZD7, FOXO3, CLCN4, LOC613316, TMTC1, WSCD1, DPYSL3, NUAK1, MLEC, EGR1, PPARGC1B, SAMD8, GFOD1, NR3C2, UHMK1, EHHADH, KIAA1671, GNAQ, LDHD, THUMPD1, SMAD3, BTG2, CHM, NRP2, NT5DC3, TP53INP2, ALCAM, BMT2, LOC781298, ZNF629, MCCC2, TMTC3, LOC101905254, KIAA1522, GDF11, PARD3B, MFSD4A, FREM2* | 76 |
| M4 | *MYBPH, SLC25A25, BOLA-DQA2, MYH4, NR1D1, MICAL2, COTL1, PTPRC, MRM2, SIRPA, UBD, LCP1, ATP2C2, SRGN, SDS, FAM229B, LOC508666, CD44, C7, SLC2A3, LOC526769, ITGAL, DBP, MYH3, ITGB2, S100A4, MAP3K8, MYO1F, LOC100297676, RSPO2, C1QB, CTXN3, CD53, CD55, UNC93B1, PLEK, HCLS1, GSDMD, MPEG1, MINDY3, DOK4, ICAM1, LOC768255, VCAM1, C1QA, ZAP70, BREH1, CYBA, CX3CL1, MS4A1, CFD, CCL1, LIPA, CXCL16, RILPL2, AEBP1, IL2RG, CX3CR1, NAV2, TMEM106A, SELL, RAC2, SH3BGRL, ARHGAP45, ARHGAP30, CD3E, GIMAP6, ACE, C1QC, CSF1R* | 70 |
| M5 | *LOC509283, MX1, ZNFX1, SLC16A6, IFI6, RSAD2, IDO1, UBA7, OAS1X, ISG15, MX2, HERC6, LOC101903126, CBFA2T3, IFI47, DDX58, SAMD9, LOC100139670, LOC512486, LOC507055, ZBP1, LOC112444847, IFIH1, PML, HSPH1, CRACD, C4A, IFI44L, IFITM1, PARP10, LOC513659, LOC511531, IRF7, IFIT3, GBP5, LOC107131209, USP18, RTP4, LOC100196901, LOC100337442, IFI44, CYP1A1, LOC618737, LOC781710, HERC5, SLFN11, OAS1Z, FAM184A, BOLA-DQA5, DHX58, LOC100298356, BATF2, LOC507271, LOC100337136, LONRF3, PRR32, IFIT2, BOLA-DQB, MOCOS, RNASE13, CNN1, PTPRE, LOC788334, LOC112441507, DRAM1, LOC504773, SLC22A4* | 67 |
| M6 | *COX3, ATP6, ND1, ND4, ND3, ND2, ATP8, RPS29, DUSP1, BTG1, NT5C3A, ATF3, LOC100848263, MRLN, PLAC8B, MIR133A-2, KRTCAP2, ETFRF1, ULBP11, TMEM258, RPL22L1, PON3, OGN, CCNC, TMEM167A, RNASEK, LOC784762, CBLN4, CCDC197, LOC514011, CEBPZOS, COA5, SLC36A4, MSMO1, LOC784160, CYP4V2, LSM5, MIR3064, LONRF1, LOC101902760, COQ10B, SMIM13, ZNF750, ACTR6* | 44 |
