# Supplementary material for: Effect of creep-feeding supplementation during the pre-weaning phase on gene co-expression in Longissimus thoracis muscle of F1 Angus x Nellore calves at weaning
Source: PLoS One. 2025 Dec 18;20(12):e0339043. doi: 10.1371/journal.pone.0339043 (PMC12714228; doi:10.1371/journal.pone.0339043)
Supplement: S4 Table — G1 = Group 1 (without creep-feeding), G2 = Group 2 (with creep-feeding), NES = Normalized Enrichment Score, GSEA = Gene Set Enrichment Analysis, Adjusted p-value for FDR (False Discovery Rate). (DOCX) [file pone.0339043.s007.docx]

**S4 Table. NES values and adjusted p-values generated by GSEA for each module in each group.**

| Module | G1 | | G2 | |
| --- | --- | --- | --- | --- |
|  | NES | Adjusted p-value | NES | Adjusted p-value |
| M1 | 4.05 | 3.742e-50 | -4.00 | 3.943e-51 |
| M2 | -6.16 | 6.219e-81 | 6.06 | 4.572e-80 |
| M3 | -3.55 | 1.189e-15 | 3.45 | 1.990e-15 |
| M4 | 2.30 | 4.289e-07 | -2.32 | 2.401e-07 |
| M5 | 1.35 | 0.082 | -1.36 | 0.081 |
| M6 | 1.90 | 0.001 | -1.91 | 0.001 |

G1 = Group 1 (without creep-feeding), G2 = Group 2 (with creep-feeding), NES = Normalized Enrichment Score, GSEA = Gene Set Enrichment Analysis, Adjusted p-value for FDR (False Discovery Rate)
