# Supplementary material for: Effect of creep-feeding supplementation during the pre-weaning phase on gene co-expression in Longissimus thoracis muscle of F1 Angus x Nellore calves at weaning
Source: PLoS One. 2025 Dec 18;20(12):e0339043. doi: 10.1371/journal.pone.0339043 (PMC12714228; doi:10.1371/journal.pone.0339043)
Supplement: S5 Table — (DOCX) [file pone.0339043.s008.docx]

**S5 Table. All terms from module 1 (GO Biological Process, KEGG pathways, REACTOME pathways, and WikiPathways) with adjusted p-value < 0.05.**

| **Terms** | **Adjusted p-value** | **Genes** |
| --- | --- | --- |
| **GO Biological Processes** | | |
| GO:0033993~Response to lipid | <0.001 | *TXNIP, ANKRD1, PDK4, DDIT4, STAT5B, TRIM63, UCP3, PER1, ZFP36, NFKBIA, FBXO32, RPS6KA3, CEBPB, KLF9, KLF4, ALAD, ASS1, ABCA1, HOXA9, PDE4B, UCP2, ADAM9, CDKN1A, THBS1, FOXO1, PPARD, GPX3, ZNF703, NPC1, FOSL2, METTL21C, CPT1A, RET, SOCS2, ALPL, PLSCR4* |
| GO:0031960~Response to corticosteroid | <0.001 | *DDIT4, TRIM63, UCP3, ZFP36, FBXO32, KLF9, ALAD, ASS1, UCP2, ADAM9, CDKN1A, FOSL2, METTL21C, ALPL* |
| GO:0048545~Response to steroid hormone | <0.001 | *TXNIP, DDIT4, TRIM63, UCP3, PER1, ZFP36, FBXO32, KLF9, ALAD, ASS1, UCP2, ADAM9, CDKN1A, THBS1, PPARD, NPC1, FOSL2, METTL21C, ALPL* |
| GO:0009725~Response to hormone | <0.001 | *TXNIP, PDK4, DDIT4, SORBS1, STAT5B, TRIM63, UCP3, PER1, NAMPT, ZFP36, LPIN1, FBXO32, ADIPOR2, KLF9, GSTM3, ALAD, ASS1, UCP2, ADAM9, CDKN1A, THBS1, GCNT1, FOXO1, KLF15, SIK2, PPARD, NPC1, FOSL2, METTL21C, SOCS2, ALPL* |
| GO:0014070~Response to organic cyclic compound | <0.001 | *TXNIP, DDIT4, STAT5B, TRIM63, UCP3, PER1, RIOK3, ZFP36, MTHFR, NFKBIA, FBXO32, BCL2L1, KLF9, KLF4, ALAD, ASS1, ABCA1, HOXA9, UCP2, ADAM9, CDKN1A, THBS1, PPARD, ZNF703, NPC1, FOSL2, METTL21C, CPT1A, SOCS2, ALPL* |
| GO:0019915~Lipid storage | <0.001 | *STAT5B, PNPLA2, NFKBIA, ABCA1, OSBPL11, PLIN5, PPARD, APOE, NPC1, CPT1A* |
| GO:0034440~Lipid oxidation | 0.001 | *PDK4, ADIPOR2, ACOX3, CPT1B, HACL1, PPARGC1A, MLYCD, PLIN5, PPARD, CPT1A* |
| GO:0031667~Response to nutrient levels | 0.001 | *GLUL, PDK4, UCP3, KLF10, BNIP3, ZFP36, SESN1, MTHFR, GABARAPL1, ALAD, ASS1, ABCA1, UCP2, CDKN1A, PPARGC1A, MLYCD, FOXO1, PPARD, APOE, CPT1A, ALPL* |
| GO:1901654~Response to ketone | 0.001 | *TXNIP, DDIT4, UCP3, FBXO32, BCL2L1, KLF9, KLF4, ASS1, HOXA9, UCP2, CDKN1A, THBS1, METTL21C* |
| GO:0045444~Fat cell differentiation | 0.002 | *BNIP3, ZFP36, ARID5B, CEBPB, ZBTB16, KLF4, TMEM120B, CEBPD, OSBPL11, PPARGC1A, FOXO1, PPARD, METRNL, FOSL2* |
| GO:0044282~Small molecule catabolic process | 0.002 | *GLUL, XDH, SCARF1, LPIN1, ACOX3, CPT1B, DDO, HACL1, MLYCD, PLIN5, PPARD, APOE, TST, CPT1A, PM20D2, FAH, CBR3* |
| GO:0010883~Regulation of lipid storage | 0.003 | *PNPLA2, NFKBIA, ABCA1, OSBPL11, PLIN5, PPARD, CPT1A* |
| GO:0046320~Regulation of fatty acid oxidation | 0.005 | *PDK4, PPARGC1A, MLYCD, PLIN5, CPT1A* |
| GO:0072593~Reactive oxygen species metabolic process | 0.005 | *PDK4, DDIT4, XDH, BNIP3, SESN1, CCN1, GADD45A, UCP2, CDKN1A, THBS1, PLIN5, FOXO1, GPX3* |
| GO:0045834~Positive regulation of lipid metabolic process | 0.005 | *SORBS1, PNPLA2, CCN1, PPARGC1A, MLYCD, PLIN5, PPARD, APOE, CPT1A* |
| GO:0016054~Organic acid catabolic process | 0.005 | *GLUL, LPIN1, ACOX3, CPT1B, DDO, HACL1, MLYCD, PLIN5, PPARD, TST, CPT1A, PM20D2, FAH* |
| GO:0070542~Response to fatty acid | 0.005 | *PDK4, ALAD, ASS1, UCP2, FOXO1, CPT1A* |
| GO:0009410~Response to xenobiotic stimulus | 0.005 | *TXNIP, ANKRD1, MTHFR, GSTM3, ALAD, ASS1, ABCA1, PDE4B, CDKN1A, THBS1, NPC1, FOSL2, CPT1A, RET, MYC, CBR3* |
| GO:0043068~Positive regulation of programmed cell death | 0.006 | *TXNIP, ANKRD1, JUN, BNIP3, RAPSN, CCN1, ZBTB16, GADD45A, TNFRSF12A, RGCC, KLF11, SIK1, CDKN1A, THBS1, GADD45G, FOXO1, VNN1, HMOX1, HTATIP2, RET, MYC* |
| GO:0071396~Cellular response to lipid | 0.007 | *ANKRD1, PDK4, DDIT4, PER1, ZFP36, NFKBIA, FBXO32, KLF9, KLF4, ASS1, ABCA1, PDE4B, ADAM9, PPARD, ZNF703, NPC1, METTL21C, CPT1A, RET, PLSCR4* |
| GO:0072329~Monocarboxylic acid catabolic process | 0.007 | *LPIN1, ACOX3, CPT1B, HACL1, MLYCD, PLIN5, PPARD, CPT1A, FAH* |
| GO:0045923~Positive regulation of fatty acid metabolic process | 0.008 | *PPARGC1A, MLYCD, PLIN5, PPARD, CPT1A* |
| GO:0019433~Triglyceride catabolic process | 0.008 | *PNPLA2, MGLL, PLIN5, LIPE* |
| GO:0006641~Triglyceride metabolic process | 0.008 | *PNPLA2, MGLL, LPIN1, SIK1, PLIN5, APOE, CPT1A, LIPE* |
| GO:0030855~Epithelial cell differentiation | 0.008 | *TXNIP, RILPL1, XDH, STAT5B, ZFP36, CEBPB, HSF4, KLF4, GSTM3, ADAM9, CDKN1A, KLF15, APOLD1, ZNF703, ATOH8, TST, FOSL2, TPRN, CPT1A, TBX3* |
| GO:0071385~Cellular response to glucocorticoid stimulus | 0.008 | *DDIT4, ZFP36, FBXO32, KLF9, ASS1, METTL21C* |
| GO:0009062~Fatty acid catabolic process | 0.008 | *LPIN1, ACOX3, CPT1B, HACL1, MLYCD, PLIN5, PPARD, CPT1A* |
| GO:0010038~Response to metal ion | 0.008 | *TXNIP, JUN, BNIP3, ALAD, ASS1, UCP2, ADAM9, THBS1, UROS, HMOX1, NPC1, MT2A, IMPA2, KCNMB4* |
| GO:0031099~Regeneration | 0.008 | *MUSTN1, XIRP1, SCARF1, LPIN1, CEBPB, KLF4, UCP2, CDKN1A, PPARD, CPT1A, PTPN3* |
| GO:0071384~Cellular response to corticosteroid stimulus | 0.009 | *DDIT4, ZFP36, FBXO32, KLF9, ASS1, METTL21C* |
| GO:0010035~Response to inorganic substance | 0.009 | *TXNIP, JUN, UCP3, BNIP3, KLF4, ALAD, ASS1, UCP2, ADAM9, THBS1, UROS, FOXO1, HMOX1, NPC1, MT2A, IMPA2, ALPL, KCNMB4* |
| GO:0070482~Response to oxygen levels | 0.009 | *DDIT4, UCP3, BNIP3, MTHFR, ALAD, RGCC, UCP2, CDKN1A, THBS1, FOXO1, PPARD, ANGPTL4, FOSL2, CPT1A, MYC* |
| GO:0006979~Response to oxidative stress | 0.009 | *TXNIP, JUN, UCP3, BNIP3, SESN1, KLF4, ALAD, UCP2, ADAM9, PPARGC1A, NAPRT, FOXO1, APOE, GPX3, VNN1, HMOX1, PTPRK* |
| GO:0034616~Response to laminar fluid shear stress | 0.009 | *KLF4, ASS1, ABCA1, ADAM9* |
| GO:0016042~Lipid catabolic process | 0.010 | *PNPLA2, SCARF1, MGLL, LPIN1, ACOX3, CPT1B, HACL1, MLYCD, PLIN5, PPARD, APOE, CPT1A, LIPE* |
| GO:0010876~Lipid localization | 0.010 | *STAT5B, UCP3, PNPLA2, NFKBIA, CPT1B, ABCA1, OSBPL11, UCP2, THBS1, PLIN5, PPARD, APOE, APOLD1, NPC1, CPT1A, PLSCR4* |
| GO:0032870~Cellular response to hormone stimulus | 0.011 | *PDK4, DDIT4, SORBS1, STAT5B, UCP3, PER1, NAMPT, ZFP36, LPIN1, FBXO32, ADIPOR2, KLF9, ASS1, UCP2, FOXO1, SIK2, PPARD, NPC1, METTL21C, SOCS2* |
| GO:0030258~Lipid modification | 0.014 | *PDK4, ADIPOR2, ACOX3, CPT1B, HACL1, PPARGC1A, MLYCD, PLIN5, PPARD, CPT1A* |
| GO:0044242~Cellular lipid catabolic process | 0.014 | *PNPLA2, MGLL, LPIN1, ACOX3, CPT1B, HACL1, MLYCD, PLIN5, PPARD, CPT1A, LIPE* |
| GO:0032787~Monocarboxylic acid metabolic process | 0.014 | *PDK4, DDIT4, TRIM63, UCP3, MGLL, LPIN1, ADIPOR2, ACOX3, CPT1B, HACL1, UCP2, PPARGC1A, MLYCD, PLIN5, PPARD, VNN1, NPC1, CPT1A, ME3, FAH* |
| GO:0031100~Animal organ regeneration | 0.014 | *LPIN1, CEBPB, UCP2, CDKN1A, CPT1A, PTPN3* |
| GO:0002237~Response to molecule of bacterial origin | 0.015 | *ANKRD1, ZFP36, NFKBIA, RPS6KA3, CEBPB, ALAD, ASS1, ABCA1, PDE4B, ADAM9, FOSL2, ALPL, PLSCR4* |
| GO:0009617~Response to bacterium | 0.015 | *ANKRD1, FKBP5, BNIP3, ZFP36, NFKBIA, RPS6KA3, CEBPB, CHD7, ALAD, ASS1, ABCA1, PDE4B, IL6R, ADAM9, FOSL2, LGALS4, ALPL, PLSCR4* |
| GO:0000302~Response to reactive oxygen species | 0.015 | *TXNIP, JUN, UCP3, BNIP3, SESN1, KLF4, UCP2, ADAM9, APOE, HMOX1, PTPRK* |
| GO:0050680~Negative regulation of epithelial cell proliferation | 0.015 | *XDH, KLF9, RGCC, THBS1, PPARD, APOE, ATOH8, PTPRK* |
| GO:0001937~Negative regulation of endothelial cell proliferation | 0.016 | *XDH, RGCC, THBS1, APOE, ATOH8* |
| GO:1905953~Negative regulation of lipid localization | 0.016 | *PNPLA2, NFKBIA, ABCA1, THBS1, PPARD* |
| GO:0010888~Negative regulation of lipid storage | 0.017 | *PNPLA2, NFKBIA, ABCA1, PPARD* |
| GO:0006638~Neutral lipid metabolic process | 0.017 | *PNPLA2, MGLL, LPIN1, SIK1, PLIN5, APOE, CPT1A, LIPE* |
| GO:0071548~Response to dexamethasone | 0.018 | *DDIT4, FBXO32, ASS1, UCP2, METTL21C* |
| GO:0046461~Neutral lipid catabolic process | 0.020 | *PNPLA2, MGLL, PLIN5, LIPE* |
| GO:0043616~Keratinocyte proliferation | 0.020 | *ZFP36, KLF9, CDKN1A, PPARD, PTPRK* |
| GO:0009409~Response to cold | 0.020 | *UCP3, NFKBIA, UCP2, FOXO1, METRNL* |
| GO:0062013~Positive regulation of small molecule metabolic process | 0.022 | *SORBS1, PPARGC1A, MLYCD, PLIN5, FOXO1, PPARD, APOE, CPT1A* |
| GO:0043537~Negative regulation of blood vessel endothelial cell migration | 0.023 | *KLF4, GADD45A, RGCC, THBS1, APOE* |
| GO:0006635~Fatty acid beta oxidation | 0.023 | *ACOX3, CPT1B, MLYCD, PLIN5, PPARD, CPT1A* |
| GO:0009266~Response to temperature stimulus | 0.023 | *UCP3, NFKBIA, TCIM, UCP2, CDKN1A, THBS1, FOXO1, HMOX1, METRNL* |
| GO:0097421~Liver regeneration | 0.024 | *CEBPB, UCP2, CPT1A, PTPN3* |
| GO:0120162~Positive regulation of cold induced thermogenesis | 0.025 | *LPIN1, CEBPB, ADIPOR2, UCP2, PPARGC1A, GADD45G, ALPL* |
| GO:0015909~Long chain fatty acid transport | 0.025 | *CPT1B, UCP2, THBS1, APOE, CPT1A* |
| GO:0061008~Hepaticobiliary system development | 0.027 | *ARID5B, CEBPB, ASS1, UCP2, NPC1, CPT1A, TBX3, PTPN3* |
| GO:0071549~Cellular response to dexamethasone stimulus | 0.028 | *DDIT4, FBXO32, ASS1, METTL21C* |
| GO:0046686~Response to cadmium ion | 0.028 | *JUN, ALAD, HMOX1, NPC1, MT2A* |
| GO:0048511~Rhythmic process | 0.028 | *STAT5B, KLF10, PER1, NAMPT, KLF9, ASS1, SIK1, PPARGC1A, ENOX2, CIART, CREM* |
| GO:1905952~Regulation of lipid localization | 0.028 | *PNPLA2, NFKBIA, ABCA1, OSBPL11, THBS1, PLIN5, PPARD, APOE* |
| GO:0050673~Epithelial cell proliferation | 0.028 | *XDH, ZFP36, BCL2L1, CEBPB, KLF9, RGCC, CDKN1A, THBS1, PPARD, APOE, HMOX1, ZNF703, ATOH8, PTPRK, MYC* |
| GO:0015908~Fatty acid transport | 0.028 | *CPT1B, UCP2, THBS1, PPARD, APOE, CPT1A* |
| GO:0019216~Regulation of lipid metabolic process | 0.031 | *PDK4, SORBS1, STAT5B, PNPLA2, CCN1, SIK1, PPARGC1A, MLYCD, PLIN5, PPARD, APOE, CPT1A* |
| GO:0019217~Regulation of fatty acid metabolic process | 0.031 | *PDK4, PPARGC1A, MLYCD, PLIN5, PPARD, CPT1A* |
| GO:0040014~Regulation of multicellular organism growth | 0.033 | *STAT5B, CHD7, FOXS1, FOSL2, SOCS2* |
| GO:0045638~Negative regulation of myeloid cell differentiation | 0.033 | *STAT5B, ZFP36, NFKBIA, ZBTB16, HOXA9, MYC* |
| GO:0051235~Maintenance of location | 0.033 | *STAT5B, PNPLA2, FTL, NFKBIA, CHD7, ABCA1, OSBPL11, PLIN5, PPARD, APOE, NPC1, METTL21C, CPT1A* |
| GO:0007584~Response to nutrient | 0.034 | *UCP3, MTHFR, ALAD, ASS1, ABCA1, MLYCD, CPT1A, ALPL* |
| GO:0048662~Negative regulation of smooth muscle cell proliferation | 0.037 | *KLF4, CDKN1A, PPARGC1A, APOE, HMOX1* |
| GO:0009913~Epidermal cell differentiation | 0.038 | *TXNIP, ZFP36, KLF4, ADAM9, CDKN1A, FOSL2, TPRN* |
| GO:0062012~Regulation of small molecule metabolic process | 0.039 | *PDK4, DDIT4, SORBS1, TRIM63, SIK1, PPARGC1A, MLYCD, PLIN5, FOXO1, PPARD, APOE, CPT1A* |
| GO:0046685~Response to arsenic containing substance | 0.041 | *ALAD, CDKN1A, UROS, HMOX1* |
| GO:0034405~Response to fluid shear stress | 0.041 | *KLF4, ASS1, ABCA1, ADAM9* |
| GO:0050996~Positive regulation of lipid catabolic process | 0.042 | *PNPLA2, PLIN5, CPT1A* |
| GO:0045600~Positive regulation of faT cell differentiation | 0.044 | *ZFP36, CEBPB, ZBTB16, PPARD, METRNL* |
| GO:0010906~Regulation of glucose metabolic process | 0.044 | *PDK4, SORBS1, SIK1, PPARGC1A, MLYCD, FOXO1* |
| GO:0045861~Negative regulation of proteolysis | 0.044 | *KLHL40, RPS6KA3, KLF4, ALAD, SPOCK2, THBS1, MAP1A, DNAJC1, PTPN3* |
| GO:0006631~Fatty acid metabolic process | 0.045 | *PDK4, UCP3, MGLL, LPIN1, ADIPOR2, ACOX3, CPT1B, HACL1, PPARGC1A, MLYCD, PLIN5, PPARD, CPT1A* |
| **REACTOME pathways** | | |
| bta9614085: Foxo mediated transcription | 0.005 | *TXNIP, TRIM63, FBXO32, KLF4, GADD45A, CDKN1A, PPARGC1A, FOXO1* |
| Circadian clock | 0.005 | *UBC, PER1, NAMPT, SIK1, PPARGC1A, KLF15, CPT1A, CREM* |
| bta449147: Signaling by ILs | 0.005 | *UBC, PSME4, JUN, STAT5B, NFKBIA, RPS6KA3, BCL2L1, CEBPD, IL32, CISH, IL6R, CDKN1A, FOXO1, HMOX1, MYC, SOCS2, MAOA, CNTFR, CLCF1* |
| bta6785807: IL 4 and IL 13 signaling | 0.009 | *BCL2L1, CEBPD, IL6R, CDKN1A, FOXO1, HMOX1, MYC, MAOA* |
| bta1280215: Cytokine signaling in immune system | 0.032 | *UBC, PSME4, JUN, STAT5B, NFKBIA, RPS6KA3, BCL2L1, CEBPD, IL32, CISH, TNFRSF12A, EIF4E3, IL6R, CDKN1A, TRIM45, FOXO1, HMOX1, MT2A, MYC, SOCS2, MAOA, CNTFR, CLCF1* |
| bta9617828: Foxo mediated transcription of cell cycle genes | 0.032 | *KLF4, GADD45A, CDKN1A, FOXO1* |
| **KEGG pathways** | | |
| bta03320: Ppar signaling pathway | 0.024 | *UBC, SORBS1, ACOX3, CPT1B, PPARD, ANGPTL4, CPT1A* |
| bta04630: Jak stat signaling pathway | 0.036 | *STAT5B, BCL2L1, CISH, IL6R, MYC, SOCS2, CNTFR, CLCF1* |
| **Wikipathways** | | |
| WP987: Adipogenesis | <0.001 | *STAT5B, NAMPT, LPIN1, CEBPB, CEBPD, GADD45A, CDKN1A, PPARGC1A, FOXO1, KLF15, PPARD, CNTFR, LIPE* |
| WP5342: Pathophysiological roles of dux4 in fshd1 | <0.001 | *TRIM63, FBXO32, CDKN1A, PPARGC1A, RET, MYC* |
| WP2882: Nuclear receptors metapathway | <0.001 | *ANKRD1, PDK4, JUN, FKBP5, FTL, GSTM3, PDE4B, PPARGC1A, FOXO1, PPARD, ANGPTL4, GPX3, HMOX1, CPT1A, MYC, CBR3* |
| WP3599: Transcription factor regulation in adipogenesis | <0.001 | *LPIN1, CEBPB, CEBPD, PPARGC1A, FOXO1* |
| WP4149: White fat cell differentiation | 0.002 | *STAT5B, CEBPB, KLF4, CEBPD, FOXO1, KLF15* |
| WP5474: Catabolism of skeletal muscle in cachexia | 0.006 | *TRIM63, NFKBIA, FBXO32, CEBPB, TNFRSF12A, IL6R* |
| WP3594: Circadian rhythm genes | 0.007 | *UBC, JUN, KLF10, PER1, NAMPT, KLF9, SIK1, PPARGC1A, CPT1A, CIART, CREM* |
| WP1403: Amp activated protein kinase signaling | 0.008 | *EIF4EBP1, PFKFB3, ADIPOR2, CPT1B, CDKN1A, CPT1A, LIPE* |
| WP706: Sudden infant death syndrome sids susceptibility pathways | 0.008 | *JUN, CEBPB, IL6R, PPARGC1A, CPT1A, RET, SCN3B, MAOA, CREM* |
| WP5102: Familial partial lipodystrophy | 0.009 | *STAT5B, PNPLA2, MGLL, KLF9, LIPE* |
| WP3942: PPAR signaling | 0.023 | *SORBS1, ACOX3, CPT1B, PPARD, ANGPTL4, CPT1A* |
| WP2037: Prolactin signaling | 0.024 | *JUN, STAT5B, EIF4EBP1, NFKBIA, CISH, MYC, SOCS2* |
| WP4321: Thermogenesis | 0.024 | *PNPLA2, MGLL, RPS6KA3, CPT1B, PPARGC1A, CREB5, CPT1A, LIPE* |
| WP2203: Thymic stromal lymphopoietin tslp signaling | 0.024 | *STAT5B, EIF4EBP1, NFKBIA, CISH, MYC* |
| WP5358: Jakstat signaling in the regulation of beta cells | 0.028 | *STAT5B, CISH, IL6R, SOCS2* |
| WP3646: Hepatitis c and hepatocellular carcinoma | 0.029 | *JUN, BCL2L1, IL6R, CDKN1A, MYC* |
| WP4659: Gastrin signaling | 0.029 | *JUN, EIF4EBP1, NFKBIA, BCL2L1, KLF4, CDKN1A, FOXO1, MYC* |
| WP2817: Mammary gland development pregnancy and lactation stage 3 of 4 | 0.029 | *TTC9, BCL2L1, CEBPB, MYC* |
| WP1050:Oxidative stress response | 0.033 | *XDH, GPX3, HMOX1, MAOA* |
| WP4658: Small cell lung cancer | 0.033 | *NFKBIA, MAX, BCL2L1, GADD45A, CDKN1A, GADD45G, MYC* |
| WP3298: Melatonin metabolism and effects | 0.043 | *PER1, FOXO1, APOE, MAOA* |
| WP1742: Tp53 network | 0.043 | *GADD45A, CDKN1A, MYC* |
| WP3274: Leptin signaling | 0.043 | *STAT5B, EIF4EBP1, BCL2L1, CISH, FOXO1, SOCS2* |
| WP1541: Energy metabolism | 0.043 | *UCP3, UCP2, PPARGC1A, FOXO1, PPARD* |
| WP2516: Atm signaling | 0.043 | *JUN, NFKBIA, GADD45A, CDKN1A* |
| WP 143: Fatty acid betaoxidation | 0.049 | *PNPLA2, CPT1B, CPT1A, LIPE* |
