# Supplementary material for: Effect of creep-feeding supplementation during the pre-weaning phase on gene co-expression in Longissimus thoracis muscle of F1 Angus x Nellore calves at weaning
Source: PLoS One. 2025 Dec 18;20(12):e0339043. doi: 10.1371/journal.pone.0339043 (PMC12714228; doi:10.1371/journal.pone.0339043)
Supplement: S6 Table — (DOCX) [file pone.0339043.s009.docx]

**S6 Table. All terms from module 2 (GO Biological Process, KEGG pathways, REACTOME pathways, and WikiPathways) with adjusted p-value < 0.05.**

| **Terms** | **Adjusted p-value** | **Genes** |  |
| --- | --- | --- | --- |
| **GO Biological Processes** | | | |
| GO:0003012~Muscle system process | 0.004 | *ACTA1, MYL1, MYH2, MYL6B, MYL3, SCN4B, LMCD1, IGFBP5, CASQ2, CHRNE, P2RY1, GRIP2, NPNT, GATM, SPX, COL14A1, KIT* |  |
| GO:0003013~Circulatory system process | 0.009 | *MYL3, SCN4B, SLC2A4, CASQ2, SLC12A2, SLC6A1, ATP2B2, P2RY1, GRIP2, P2RY2, ACTC1, SPX, POPDC2, ATP1A4, SLC4A4, HRH2, NPR3* |  |
| GO:0045444~FaT cell differentiation | 0.009 | *SLC2A4, LPL, FABP3, FRZB, DHRS7B, DUSP10, C1QTNF3, MAFB, INHBB, ZNF385A, INSIG1* |  |
| GO:0014074~Response to purine containing compound | 0.016 | *AQP1, IGFBP5, CASQ2, SLC6A1, P2RY1, P2RY2, INHBB, SRD5A1* |  |
| GO:0033275~Actin myosin filament sliding | 0.018 | *MYL1, MYH2, MYL6B, ACTC1* |  |
| GO:0006936~Muscle contraction | 0.029 | *ACTA1, MYL1, MYH2, MYL6B, MYL3, SCN4B, CASQ2, CHRNE, GRIP2, NPNT, SPX, KIT* |  |
| GO:0035725~Sodium ion transmembrane transport | 0.029 | *SCN4B, SLC9A5, SLC12A2, SLC6A1, TPCN1, ATP1A4, SLC4A4* |  |
| GO:0045229~ExteRNAl encapsulating structure organization | 0.045 | *COL15A1, CCDC80, NPNT, ADAMTS20, LUM, FMOD, COL11A2, COL14A1, ADAMTS8, COL27A1, OLFML2B* |  |
| **REACTOME pathways** | | | |
| bta397014: Muscle contraction | <0.001 | *ACTA1, MYL1, MYL6B, MYL3, CAMK2A, SCN4B, CASQ2, ATP2B2, ACTC1, CACNA2D2, CACNG4, ATP1A4* |  |
| bta382551: Transport of small molecules | 0.007 | *CAMK2A, SLC2A4, AQP1, LPL, SLC16A3, SLC9A5, CASQ2, CA2, SLC12A2, SLC6A1, ATP2B2, CYB5R2, AQP4, TPCN1, ATP1A4, GPIHBP1, SLC2A12, SLC4A4* |  |
| bta5576891: Cardiac conduction | 0.014 | *CAMK2A, SCN4B, CASQ2, ATP2B2, CACNA2D2, CACNG4, ATP1A4* |  |
| bta2022090: Assembly of collagen fibrils and other multimeric structures | 0.014 | *COL15A1, COL11A2, COL14A1, COL27A1, LAMA3* |  |
| bta 8957322: Metabolism of steroids | 0.014 | *SCD, FASN, LSS, INSIG1, SRD5A1, CYP51A1, DHCR24* |  |
| bta8948216: Collagen chain trimerization | 0.014 | *COL15A1, COL11A2, COL14A1, COL27A1* |  |
| bta5576892: Phase 0 rapid depolarisation | 0.014 | *CAMK2A, SCN4B, CACNA2D2, CACNG4* |  |
| bta1655829: Regulation of cholesterol biosynthesis by srebp srebf | 0.016 | *SCD, FASN, LSS, INSIG1, CYP51A1* |  |
| bta1474290: Collagen formation | 0.030 | *COL15A1, COL11A2, COL14A1, COL27A1, LAMA3* |  |
| bta390522: Striated muscle contraction | 0.036 | *ACTA1, MYL1, MYL3, ACTC1* |  |
| bta425407: Slc mediated transmembrane transport | 0.037 | *SLC2A4, SLC16A3, SLC9A5, SLC12A2, SLC6A1, SLC2A12, SLC4A4* |  |
| bta1655829: Activation of gene expression by srebf srebp | 0.043 | *SCD, FASN, LSS, CYP51A1* |  |
| bta1474244: Extracellular matrix organization | 0.046 | *COL15A1, ITGB6, LUM, FMOD, COL11A2, COL14A1, ADAMTS8, COL27A1, LAMA3* |  |
| **KEGG pathways** | | | |
| bta04964: Proximal tubule bicarbonate reclamation | 0.007 | *AQP1, CA2, ATP1A4, SLC4A4* |  |
| bta00100: Steroid biosynthesis | 0.027 | *LSS, CYP51A1, DHCR24* |  |
| bta01040: Biosynthesis of unsaturated fatty acids | 0.028 | *SCD, FADS2, FADS1* |  |
| bta04260: Cardiac muscle contraction | 0.028 | *MYL3, ACTC1, CACNA2D2, CACNG4, ATP1A4* |  |
| bta05410: Hypertrophic cardiomyopathy hcm | 0.045 | *MYL3, ACTC1, ITGB6, CACNA2D2, CACNG4* |  |
| bta05414: Dilated cardiomyopathy | 0.046 | *MYL3, ACTC1, ITGB6, CACNA2D2, CACNG4* |  |
| **Wikipathways** | | | |
| WP4718: Cholesterol metabolism with bloch and kandutschrussell pathways | <0.001 | *SCD, FASN, FADS2, FADS1, LSS, CYP51A1, DHCR24* |  |
| WP5314: Lactate shuttle in glial cells | 0.001 | *LDHA, SLC16A3, CA2, SLC4A4* |  |
| WP4724: Omega9 fatty acid synthesis | 0.001 | *SCD, FASN, FADS2, FADS1* |  |
| WP3114: Cori cycle | 0.002 | *LDHA, PGK1, SLC2A4, GPT* |  |
| WP5329: Cholesterol biosynthesis pathway in hepatocytes | 0.002 | *SCD, FADS2, CYB5R2, LSS, SRD5A1, CYP51A1, DHCR24* |  |
| WP3194: Sterol regulatory elementbinding proteins srebp signaling | 0.010 | *SCD, LPL, FASN, LSS, INSIG1, CYP51A1* |  |
| WP4628: Aerobic glycolysis augmented | 0.029 | *LDHA, PGK1, GPT* |  |
| WP2882: Nuclear receptors metapathway | 0.031 | *SLC2A4, SCD, BHLHE40, SLC6A1, FASN, CDC42EP3, ESR1, ACKR3, SLC2A12* |  |
| WP969: Striated muscle contraction pathway | 0.033 | *ACTA1, MYL1, MYL3, ACTC1* |  |
| WP5193: Cholesterol synthesis disorders | 0.039 | *LSS, CYP51A1, DHCR24* |  |
