# Supplementary material for: Effect of creep-feeding supplementation during the pre-weaning phase on gene co-expression in Longissimus thoracis muscle of F1 Angus x Nellore calves at weaning
Source: PLoS One. 2025 Dec 18;20(12):e0339043. doi: 10.1371/journal.pone.0339043 (PMC12714228; doi:10.1371/journal.pone.0339043)
Supplement: S7 Table — (DOCX) [file pone.0339043.s010.docx]

**S7 Table. All terms from module 3 (GO Biological Process, KEGG pathways, REACTOME pathways, and WikiPathways) with adjusted p-value < 0.05.**

| **Terms** | **Adjusted p-value** | **Genes** |  |
| --- | --- | --- | --- |
| **GO Biological Processes** | | | |
| GO:0060537~Muscle tissue development | 0.003 | *XIRP2, CSRP3, PLN, AKAP6, HLF, DLL4, SOX6, PPARA, FZD7, EGR1, SMAD3, BTG2* |  |
| GO:0007507~Heart development | 0.003 | *XIRP2, CSRP3, HSPB7, PLN, AKAP6, PTPN11, DLL4, SOX6, PPARA, FZD7, SMAD3, NRP2, FREM2* |  |
| GO:0014706~Striated muscle tissue development | 0.003 | *XIRP2, CSRP3, PLN, AKAP6, DLL4, SOX6, PPARA, FZD7, SMAD3* |  |
| GO:0061061~Muscle structure development | 0.006 | *XIRP2, CSRP3, ANKRD2, AKAP6, HLF, HBEGF, DLL4, SOX6, PPARA, SORT1, FZD7, EGR1, SMAD3, BTG2* |  |
| GO:0007517~Muscle organ development | 0.006 | *XIRP2, CSRP3, ANKRD2, HLF, HBEGF, DLL4, SOX6, EGR1, SMAD3, BTG2* |  |
| GO:0045596~Negative regulation of cell differentiation | 0.021 | *ANKRD2, PTPN11, HOOK3, SOX6, PPARA, SORT1, FZD7, FOXO3, SMAD3, BTG2, GDF11* |  |
| GO:0009725~Response to hormone | 0.021 | *ZNF106, CSRP3, PADI2, PTPN11, PRKAA2, PPARA, SORT1, FOXO3, EGR1, PPARGC1B, NR3C2, SMAD3, BTG2* |  |
| GO:0032330~Regulation of chondrocyte differentiation | 0.030 | *PTPN11, SOX6, CCN2, SMAD3* |  |
| GO:0048511~Rhythmic process | 0.031 | *HLF, NRIP1, PRKAA2, PPARA, FOXO3, EGR1, GNAQ* |  |
| **Wikipathways** | | |  |
| WP1541: Energy metabolism | 0.002 | *PRKAB2, PRKAA2, PPARA, FOXO3, PPARGC1B* |  |
| WP4540: Hippo signaling regulation | 0.015 | *PRKAR2A, PRKAB2, PRKAA2, GNAQ, SMAD3* |  |
| WP5036: Angiotensin ii receptor type 1 pathway | 0.015 | *PTPN11, CCN2, SMAD3* |  |
| WP34: Ovarian infertility | 0.015 | *NRIP1, EGR1, SMAD3* |  |
| WP304: Kit receptor signaling | 0.015 | *GAB2, PTPN11, MITF, FOXO3* |  |
| WP2571: Polycystic kidney disease pathway | 0.015 | *PRKAB2, PRKAA2, FZD7, GNAQ* |  |
| WP2380: Brainderived neurotrophic factor bdnf signaling | 0.018 | *PTPN11, PRKAA2, SORT1, FOXO3, EGR1* |  |
| WP3965: Lipid metabolism pathway | 0.018 | *PRKAR2A, PRKAB2, PRKAA2* |  |
| WP5094: Orexin receptor pathway | 0.032 | *PTPN11, HBEGF, CCN2, EGR1, GNAQ* |  |
| WP3594: Circadian rhythm genes | 0.032 | *NRIP1, PRKAA2, PPARA, EGR1, GNAQ* |  |
| WP49: Il2 signaling | 0.032 | *GAB2, PTPN11, FOXO3* |  |
| WP5353: acrophagestimulating protein msp signaling | 0.037 | *HK2, PRKAA2, EGR1, SMAD3* |  |
| WP2895: Differentiation of white and brown adipocyte | 0.044 | *HSPB7, PPARGC1B* |  |
| WP5318: Female steroid hormones in cardiomyocyte energy metabolism | 0.045 | *PPARA, PPARGC1B* |  |
| WP5382: Tgfb smad signaling | 0.045 | *CCN2, SMAD3* |  |
| WP4239: Epithelial to mesenchymal transition in colorectal cancer | 0.045 | *DLL4, FZD7, SMAD3, NRP2* |  |
| WP4659: Gastrin signaling | 0.045 | *PTPN11, FOXO3, EGR1, GNAQ* |  |
| WP3888: Vegfavegfr2 signaling | 0.045 | *PTPN11, HBEGF, DLL4, PRKAA2, CCN2, FOXO3, EGR1, NRP2* |  |
